# First-Principles Calculations of Thermoelectric Transport Properties of Quaternary and Ternary Bulk Chalcogenide Crystals

**DOI:** 10.3390/ma15082843

**Published:** 2022-04-13

**Authors:** Sahib Hasan, Saro San, Khagendra Baral, Neng Li, Paul Rulis, Wai-Yim Ching

**Affiliations:** 1 Department of Physics and Astronomy, University of Missouri-Kansas City, Kansas City, MO 64110, USA; sahmvd@mail.umkc.edu (S.H.); ssawcc@mail.umkc.edu (S.S.); kbx67@mail.umkc.edu (K.B.); rulisp@umkc.edu (P.R.); 2 Department of Sciences, College of Basic Education, Al Muthanna University, Samawah 66001, Iraq; 3 School of Materials Science and Engineering, Wuhan University of Technology, No. 122, Luoshi Road, Wuhan 430070, China; lineng@whut.edu.cn

**Keywords:** chalcogenide crystals, Boltzmann theory, Seebeck coefficient, thermoelectric properties, total bond order density, density functional theory

## Abstract

Chalcogenide crystals have a wide range of applications, especially as thermoelectric materials for energy conversion. Thermoelectric materials can be used to generate an electric current from a temperature gradient based on the Seebeck effect and based on the Peltier effect, and they can be used in cooling applications. Using first-principles calculations and semiclassical Boltzmann theory, we have computed the Seebeck coefficient, electrical conductivity, electronic thermal conductivity, power factor, and figure of merit of 30 chalcogenide crystals. A Quantum Espresso package is used to calculate the electronic properties and locate the Fermi level. The transport properties are then calculated using the BoltzTraP code. The 30 crystals are divided into two groups. The first group has four crystals with quaternary composition (*A*_2_*BCQ*_4_) (A = Tl; B = Cd, Hg; C = Si, Ge, Sn; Q = S, Se, Te). The second group contains 26 crystals with the ternary composition (*A’B’Q*_2_) (A’ = Ag, Cu, Au, Na; B’ = B, Al, Ga, In; Q = S, Se, Te). Among these 30 chalcogenide crystals, the results for 11 crystals: Tl_2_CdGeSe_4_, Tl_2_CdSnSe_4_, Tl_2_HgSiSe_4_, Tl_2_HgSnS_4_, AuBSe_2_, AuBTe_2_, AuAlTe_2_, AuGaTe_2_, AuInTe_2_, AgAlSe_2_, and AgAlTe_2_ are revealed for the first time. In addition, temperature-dependent transport properties of pure and doped AgSbSe_2_ and AgSbTe_2_ crystals with dopant compositions of AgSb_0.94_Cd_0.06_Te_2_ and AgSbTe_1.85_Se_0.15_ were explored. These results provide an excellent database for bulk chalcogenides crucial for a wide range of potential applications in renewable energy fields.

## 1. Introduction

In recent years, searching for new alternative renewable energy resources has become an essential endeavor due to severe environmental concerns and the concomitant need to reduce the use of fossil fuels [1,2,3]. This is especially true for materials that can convert energy that would otherwise go to waste into useful work. The direct conversion between heat and electricity is crucial for many applications in power generation [4,5,6,7]. In this regard, thermoelectric (TE) materials have attracted a great deal of attention because of their ability to convert the waste heat from industrial processes into usable electricity [8]. The TE effect was first noticed by Seebeck in 1821 and was coined as the Peltier effect when used to create a temperature gradient from a supplied current [9]. This method of energy generation was used in spacecraft beginning in the early 1960s [10,11]. Since then, exploring high-efficiency thermoelectric materials for clean and renewable energy production has become one of the top priorities in materials research and development. Even though many TE devices have low efficiency, they still have been widely used in many applications for energy harvesting. They are expected to have broad and diverse applications to the recovery of waste heat for useful work, as illustrated, for example, by the wristwatch design of the Seiko and Citizen companies [12,13].

The overall performance of a TE material is measured by a dimensionless figure of merit parameter *ZT*, given by $ZT=S^{2}\sigma T/\left(\kappa_{lat}+\kappa_{ele}\right)$, where *S*, *σ*, *T*, *κ_lat_*, and *κ_ele_* are the Seebeck coefficient, electrical conductivity, absolute temperature, lattice thermal conductivity, and electronic thermal conductivity, respectively. *σ* is given by σ=nem, where *n* is the carrier concentration, e is the electron charge, and m is the electron mobility. The σ and *κ_ele_* are related by $\kappa_{ele}=L_{0}\sigma T$, where *L*_0_ is the Lorenz number [14]. $S^{2}\sigma$ is called power factor (PF). For excellent TE materials, *ZT* should be larger than unity [15,16]. Many efforts have been dedicated to increase *ZT* by new innovative approaches such as using nanowires, nano-structured materials, band structure engineering, lattice anharmonicity, and two-dimensional materials technology [17,18,19,20,21,22].

Various materials are used for thermoelectricity over a wide range of temperatures, including polymeric and inorganic materials. However, inorganic semiconductors have been found to be the most efficient [23,24]. Among the inorganic materials, chalcogenide compounds have attracted a great deal of attention. They generally have high electrical conductivity and low thermal conductivity resulting in higher *ZT* values [25]. Chalcogenide compounds contain at least one of the chalcogen elements (S, Se, Te) and one or more electropositive, in a few cases, electronegative elements. The electropositive elements are mostly from group IB (Cu, Ag), IIB (Zn, Cd, Hg), IVA (Si, Ge, Sn), IIIA (In, Tl), IVB (Zr, Hf), IIA (Mg, Ba), and IA (Li, K, Cs) in the periodic table. In some cases, lanthanide elements such as La and Lu can also be involved. This diverse compositional space makes chalcogenide compounds a unique class of materials rarely seen in other materials such as semiconductors, large gap insulators, superconductors, silicates glasses, metallic alloys, etc. Many quaternary chalcogenide crystals, such as A^I^–B^III^–C^IV^–X^VI^ system (A^I^ = Cu, Ag; B^III^ = Ga, In; C^IV^ = Si, Ge, Sn; X^VI^ = S, Se, Te), and A^I^_2_B^II^C^IV^Q_4_ system (A^I^ = Cu, Ag; B^II^ = Mg, Mn, Fe, Zn, Cd, and Hg; C^IV^ = Si, Ge, Sn; and Q = S, Se, Te) are very popular due to their compositional flexibility and functional turnability ideal for TE applications [26,27,28,29,30]. They are also extensively used in optical [31] and nonlinear optical devices in the visible-infrared region, photovoltaic cells [32,33,34,35,36,37,38], solar energy converters [39,40,41,42], and magnetic applications [43]. Another group of chalcogenide crystals in the form of Ag_2_XYQ_4_ (X = Ba, Sr; Y = Ge, Sn; Q = S, Se) are also very attractive [44].

Several experimental and theoretical pieces of research have been carried out recently to enhance the thermoelectric performance and increase the *ZT* of some chalcogenide crystals. C. Wang et al. [45] has succeeded recently in achieving *ZT* higher than one for Ag-doped crystalline CuInTe_2_, where *ZT* was increased for Cu_1−x_Ag_x_InTe_2_ from 0.6 (at x = 0) to 1.4 (at x = 0.25). A very high enhanced figure of merit (*ZT* ≈ 2.6 at 573 K) was obtained in Cd-doped polycrystalline AgSbTe_2_ [46]. Another study [47] showed that *ZT* increased to maximum value (*ZT* ≈ 1.35 at 600 K) for AgSbTe_2−x_Se_x_ (at x = 0.02). S. Deng et al. [48] showed that the TE performance could be enhanced for Ga-doped CuInTe_2_ crystal (CuIn_1−x_Ga_x_Te_2_), and ZT can be increased to the maximum value (*ZT* = 0.8 at 773 K) for the CuIn_0.8_Ga_0.2_Te_2_ sample due to the lower thermal conductivity. Y. Zhong et al. [49] found that Ag vacancy and In dopant substitution in the AgGaTe_2_ system can lower the lattice thermal conductivity (*κ_lat_*) significantly, which can produce a higher value of *ZT* (*ZT* ≈ 1.44).

SnTe is another type of chalcogenide crystal that has been under extensive experimental and theoretical studies for a long time. Recently, A. Rifqi et al. [50] succeeded in enhancing the TE performance of Ti-doped SnTe crystal (Sn_1−x_Ti_x_Te) and Zr-doped SnTe crystal (Sn_1−x_Zr_x_Te). They increased *ZT* from 0.41 to 0.51 at 700 K for Sn_0.97_Ti_0.03_Te(x = 0.03), and from 0.45 to 0.55 at 700 K for Sn_0.98_Zr_0.02_Te (x = 0.02). New experimental techniques have been used recently to achieve higher *ZT* for some chalcogenides. For example, by using new mechanical alloying combined with microwave-assisted synthesis for the synthesis of single-phase cubic isocubanite CuFe_2_S_3_, a maximum thermoelectric figure of merit, *ZT*_max_ ≈ 0.14 at 673 K, was achieved for CuFe_2_S_3_ crystal [51]. Thermoelectric transport properties of another important group of chalcogenides, Tl_2_PbXY_4_ (X = Zr, Hf and Y = S, Se), have been reported recently by S. Azam et al. [52]. According to this study, these four crystals possess high TE performance and high *ZT* (*ZT* = 0.85, 0.71, 0.725, and 0.68 at 800 K for Tl_2_PbHfS_4_, Tl_2_PbHfSe_4_, Tl_2_PbZrS_4_, and Tl_2_PbZrSe_4_ crystals respectively).

The crystal structure, electronic structure, and the optical properties of quaternary chalcogenides Tl_2_B^II^C^IV^Q_4_ (B^II^ = Cd, Hg; C^IV^ = Si, Ge, Sn; Q = S, Se, Te) have been reported recently [53,54,55,56]. They are semiconductors with potential TE applications but have not yet been sufficiently explored so far. Bagci et al. [57] reported the chemical-potential-dependent transport properties of the chalcopyrite crystals CuBQ_2_ (Q = S, Se, Te). However, they did not include the temperature dependency of the transport properties. Although the TE properties of ADQ_2_ (A = Cu, Ag; D = Ga, In; Q = Se, Te) chalcopyrite were studied extensively [58,59,60,61,62,63,64], much less research has been conducted on AuBQ_2_ and AgAlQ_2_ (Q = S, Se, Te). On the other hand, the electronic structure and optical properties of AuBQ_2_ and AgAlQ_2_ were reported recently [65,66] without the TE transport properties. The TE properties of the ternary chalcogenide crystals AgSbSe_2_ and AgSbTe_2_ were studied sixty years ago in the 1950s [67]. These two crystals belong to the I-V-VI_2_ family, where I = Cu or Ag, V = Sb or Bi, and VI = S, Se, and Te. They were considered promising p-type TE materials because of their low thermal conductivity [68,69,70]. Both of these crystals can crystallize in a rock salt crystal structure (space group 225, $Fm\bar{3}m$) with disordered Ag and Sb atoms [71]. Another study [72] revealed that they are semiconductors with a very narrow energy band gap (E_g_ ≈ 0.03 eV) or that they are semi-metallic. TE performance for AgSbSe_2_ and AgSbTe_2_ can be significantly enhanced (e.g., *ZT* is greater than 1) when properly doped by Pb, Bi, Cd, Sn, Se, or Ce dopants [46,47,73,74,75,76].

Motivated by the realization of the unique properties of the above crystals, we investigate the TE transport properties of 30 chalcogenide crystals as listed in Table S1 in the Supplementary Information (SI) by using density functional theory and semiclassical Boltzmann theory. The results for these 30 crystals are presented in Section 3.1. These 30 crystals are divided into two main groups: crystals 1–4 with stoichiometry *A*_2_*BCQ*_4_ (A = Tl; B = Cd, Hg; C = Si, Ge, Sn; Q = S, Se, Te) (colored white in Table S1 and Table 1), crystals 5–30 with stoichiometry (*A’B’Q*_2_) (A = Ag, Cu, Au, Na; B’ = B, Al, Ga, In; Q = S, Se, Te). The crystals 5–30 are divided into six subgroups (colored gray and white in Table S1 and Table 1). In all subsequent discuss ions, the same specific order and ID number for these crystals are maintained. The fully optimized structures are listed in Table S1 with the corresponding experimental lattice parameters. Moreover, we further investigated the crystals AgSbSe_2_, AgSbTe_2_, Cd-doped AgSbTe_2_ (AgSb_0.94_Cd_0.06_Te_2_), and Se-doped AgSbTe_2_ (AgSbTe_1.85_Se_0.15_) by using the same computational methods, and their results are presented in Section 3.2. Such comprehensive studies have added valuable insight into chalcogenide crystal sciences and are to provide a comprehensive review for the industry. In the following section, we briefly describe the computational methods used, followed by the results and discussions section. We end up with a brief conclusion and our vision for the future study on how to improve their thermoelectric performance.

## 2. Computational Methods

In this work, two well-defined density functional theory (DFT) based methods were used for specific targeted goals, which are: (1) the Orthogonalized Linear Combination of Atomic Orbitals (OLCAO) method [77] and (2) Quantum Espresso (QE) [78]. All the calculations have been performed based on the previously studied crystals [79,80], relaxed by Vienna Ab initio Simulation Package (VASP) [81]. The Spin–Orbit Coupling (SOC) effect was not included in our DFT calculations. The OLCAO method was used to calculate the electronic structure and the interatomic bonding [77]. OLCAO is an all-electron method based on the local density approximation. It uses the atomic orbitals that are themselves expanded as Gaussian-type orbitals (GTO) on the basis of expansion of the solid-state wave function. The use of localized atomic orbitals in the basis expansion, in contrast to the plane-wave expansion, is particularly effective for both crystalline [79,82,83,84,85] and non-crystalline materials [86,87,88], especially those with complex structures typical in the biomolecular systems [89,90]. A sufficiently large number of k-points (10 × 10 × 12 for the crystals 1–4, to 16 × 16 × 8 for most crystals 5–30) were used for band structure calculations based on the size of the crystal. We used the Mulliken scheme [91] for the calculation of partial charge (PC) and interatomic bonding. The PC of an atom is defined as the charge deviation of the effective charge Q* from the charge of neutral atom (Q_0_) in units of electron charge. Mathematically, ΔQ = Q_0_ − Q*. Negative ΔQ implies a gain of electrons (i.e., an electronegative ion), and positive ΔQ implies a loss of electrons (i.e., electropositivity). Equations (1) and (2) show the formulae for effective charge ($Q_{\alpha }^{*}$) and bond order (BO) values, also called the overlap population, $\rho_{\alpha \beta }$ between any pair of atoms (*α*, *β*).

(1) $$Q_{\alpha }^{*}=\underset{i}{\sum }\underset{m,occ}{\sum }\underset{j,\beta }{\sum }C_{i\alpha }^{*m}C_{j\beta }^{m}S_{i\alpha ,j\beta }$$

(2) $$\rho_{\alpha \beta }=\underset{m,\;occ}{\sum }\underset{i,j}{\sum }C_{i\alpha }^{*m}C_{j\beta }^{m}S_{i\alpha ,j\beta }$$

In the above equations, $S_{i\alpha .j\beta }$ are the overlap integrals between the ith orbital in the *α*th atom and the *j*th orbital in *β*th atom. $C_{j\beta }^{m}$ is the eigenvector coefficients of the *m*th occupied band. The BO (Equation (2)) defines the relative strength of the bond. The summation of all BO values in the crystal gives the total bond order (TBO). We obtain the total bond order density (TBOD) when TBO is normalized by the cell volume. TBOD is a single quantum mechanical metric to describe the internal cohesion of the crystal [92].

For the thermoelectric transport properties, Quantum Espresso (QE) was used to calculate the total energy, band structure, Fermi level energy, etc. The generalized gradient approximation (GGA) of Perdew, Burke, and Ernzerhof (PBE) potential was used in QE [78,93]. QE uses plane waves as basis set that includes the scalar relativistic effects in the pseudo-potentials. In this study, the energy cut-off of the plane waves was set to be 40 Ryd (≈544 eV). In the QE calculations for the electronic and band structure for transport properties using BoltzTraP code, we needed denser k-point meshes for self-consistent calculations. Therefore, after careful convergence tests, a grid of 12 × 12 × 16 k-points was used for the crystals 1–4, while a grid of 18 × 18 × 9 k-points was found to be sufficient in most crystals 5–30 for the self-consistent calculations. The AgSbSe_2_ and AgSbTe_2_ crystals with 16 atoms in the unit cell have fully relaxed lattice parameters of a = 5.8010Å and 6.0991 Å, respectively. While a supercell with 64 atoms was created for both the Cd-doped AgSbTe2 (AgSb_0.94_Cd_0.06_Te_2_) and Se-doped AgSbTe_2_(AgSbTe_1.85_Se_0.15_) crystals, which have relaxed lattice parameters of a = 12.1977 Å and 12.2109 Å respectively. For the cubic supercell of AgSbTe_1.85_Se_0.15_, we started creating 2 × 2 × 2 supercells from the fully relaxed cubic crystal structure of AgSbTe_2_ with eight atoms. In the second step, five Te atoms, located at different and totally random sites of the supercell, were replaced by five Se atoms. The sites at the six faces of the supercell were avoided. The second step was followed by fully VASP relaxation for the supercell. Another last VASP relaxation step was performed to make sure that each atom took its normal position (that minimizes the energy) in the supercell.

A special feature in QE is the ability to calculate TE transport properties using the semiclassical Boltzmann theory within the constant scattering time approximation (CSTA) as implemented in the BoltzTraP code [94]. In this theory, the Seebeck coefficient, electrical conductivity, and electronic thermal conductivity can be expressed in the following equations [94]:

(3) $$S_{\alpha \beta }\left(T,\;\mu \right)=\;\frac{1}{eT\Omega \sigma_{\alpha \beta }\left(T,\;\mu \right)}\int \sigma_{\alpha \beta }\left(\varepsilon \right)\left(\varepsilon -\mu \right)\left[-\frac{\delta f_{0}\left(T,\;\varepsilon ,\;\mu \right)}{\delta \varepsilon }\right]d\varepsilon$$

(4) $$\sigma_{\alpha \beta }\left(T,\;\mu \right)=\;\frac{1}{\Omega }\int \sigma_{\alpha \beta }\left(\varepsilon \right)\left[-\frac{\delta f_{0}\left(T,\;\varepsilon ,\;\mu \right)}{\delta \varepsilon }\right]d\varepsilon$$

(5) $$\kappa_{\alpha \beta }\left(T,\;\mu \right)=\;\frac{1}{e^{2}T\Omega }\int \sigma_{\alpha \beta }\left(\varepsilon \right){\left(\varepsilon -\mu \right)}^{2}\left[-\frac{\delta f_{0}\left(T,\;\varepsilon ,\;\mu \right)}{\delta \varepsilon }\right]d\varepsilon$$

Here, $\sigma_{\alpha \beta }$ are the transport distribution tensor elements calculated using the Fourier interpolation of the band structure. *α* and *β* are the tensor indices; *Ω*, *μ*, *f*_0_, and *T* are the cell volume, chemical potential, Fermi distribution function, and absolute temperature, respectively.

The input data needed to run BoltzTraP code are the crystal structure and the band structure on a uniform grid. The BoltzTraP code solves the Boltzmann equation [95],

(6) $$\frac{\partial }{\partial t}f+\vec{v}\frac{\partial }{\partial \vec{r}}f+\frac{eE}{\hbar }\frac{\partial }{\partial \vec{k}}f={\left(\frac{\partial f}{\partial t}\right)}_{scattering}$$

where, *f* is the distribution function, *ν* is the velocity vector of the particle, and *k* is the wave vector.

BoltzTraP code solves the Boltzmann equation by interpolating a band structure and performing all required integrations. The BoltzTraP code has been tested over the last decade on different materials ranging from superconductors [96] to thermoelectric materials [97,98,99,100]. Good agreement with the experimental values was achieved in many cases [101,102,103]. It should be pointed out that the *ZT* values obtained here are slightly overestimated because the phonon contribution to the thermal conductivity was ignored due to the limitation of the BoltzTraP code. The BoltzTraP code treats the lattice thermal conductivity contributed by phonons as a constant and provides only the electronic part of the thermal conductivity. Several simplifying approximations are introduced. The chemical potential (*μ*) is set to the Fermi level energy (*μ_F_*) because conductivities (thermal and electronic) depend proportionally on the relaxation time τ within the constant relaxation time framework. Those values can then simply be multiplied by the constant relaxation time (10^−14^ s, as this value is used in BoltzTraP code) to obtain the final transport properties. The Seebeck coefficient does not depend on the relaxation time within the constant relaxation time approximation. The CSTA assumes that the relaxation time τ is independent of energy. Hence the power factor is τ dependent, and *ZT* is τ independent. Nevertheless, this method has successfully described the transport properties of a wide range of thermoelectric materials [94,104,105,106].

## 3. Results and Discussion

### 3.1. Transport Properties of 30 Chalcogenide Crystals

**Figure 1:**
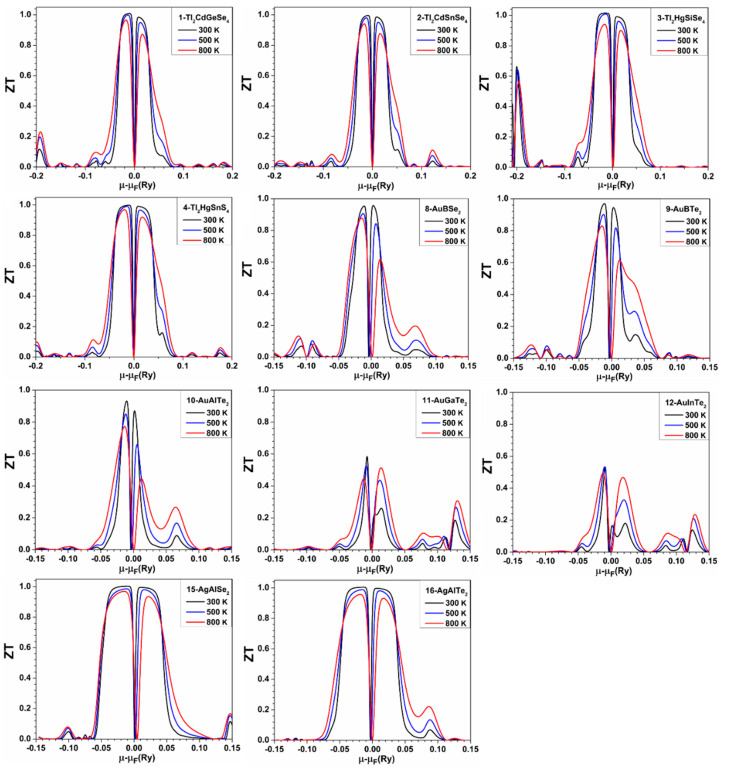
*ZT* versus the chemical potential for the crystals: 1-Tl_2_CdGeSe_4_, 2-Tl_2_CdSnSe_4_, 3-Tl_2_HgSiSe_4_, 4-Tl_2_HgSnS_4_, 8-AuBSe_2_, 9-AuBTe_2_, 10-AuAlTe_2_, 11-AuGaTe_2_, 12-AuInTe_2_, 15-AgAlSe_2_, and 16-AgAlTe_2_.

The DFT calculations for the energy band gap (*E_g_*) for some of these 30 crystals were published in our two previous works [79,80], and we have listed them in the third column of Table S2. In this work, we mostly focus on the crystals of the first group (the crystals: 1–4), and the crystals of the first (the crystals: 5–7), second (the crystals: 8–12), and third (the crystals: 13–16) subgroups of the second group (total crystals: 1–16), for which the transport properties are calculated for the first time. The calculated *S* and PF with varying ranges of *μ* for these 16 crystals at three different temperatures (300 K, 500 K, and 800 K) are shown in Figures S1 and S2 in the SI. Figures S3 and S4 show the *S* and PF versus the chemical potential for the remaining 14 crystals in the second main group. Figures S5–S7 show the electrical conductivity, electronic thermal conductivity, and the figure of merit versus the chemical potential for all 30 crystals. It is crucial to point out that all chemical-potential-dependent transport properties (including *ZT* in Figure 1) at three fixed temperatures (300 K, 500 K, and 800 K) were calculated by fixing the temperature and letting the carrier concentrations change. That means for a fixed temperature, the chemical potential is simply a function of carrier concentration, and the chemical potential *µ* can vary.

Table S2 shows the calculated values of *S* and *ZT* for these 30 crystals at room temperature (300 K). *S* can vary with the variation of the carrier concentration, which determines the value of the induced thermoelectric voltage due to the difference in temperature across the material. *S* is very sensitive to chemical potential, carrier concentration, and temperature. The positive value of *S* shows that holes have a dominant contribution to the conduction (p-type TE material), while the negative value of *S* shows that electrons have a dominant contribution to the conduction (n-type TE material). In insulators, *S* has the highest values around the Fermi energy, or μ−μF ≈ 0, because the carrier concentrations are at their lowest values. It is evidently observed from Figures S1 and S3 that *S* is significantly improved in the vicinity of μ−μF=0, which indicates that a fairly large value of *S* can be attained by small n-type or p-type doping. From Figures S1 and S3, we can also notice that the highest *S* can be found in the crystals 5-CuBS_2_, 6-CuBSe_2_, 29-NaInSe_2_, and 30-NaInTe_2_ with values: 2600, 2500, 2400, and 2100 μV/K respectively for p-type crystals. Generally, the maximum value of *S* is obtained at room temperature, 300 K (without fixing the carrier concentrations), and *S* decreases with an increase in temperature at certain values of chemical potential. Much fewer previous studies for the chemical potential transport properties of these 30 crystals were found in the literature, so we are unable to compare all our results in Table S2. S values in Table S2 may coincide with high values of electronic thermal conductivity, so it is necessary to fix the carrier concentrations when calculating *S* as a function of temperature.

Another important parameter that can determine the TE performance of a material is the power factor (PF). Figure S2 shows the PF as a function of the chemical potential at three different temperatures, 300 K, 500 K, and 800 K for crystals: 1–16, while the results for the remaining crystals are shown in Figure S4. As can be seen in these figures, the highest PF occurs at 800 K for all crystals, except for 29-NaInSe_2_, for which the highest value of PF occurs at 500 K instead of 800 K. We mentioned previously that *S* has its maximum value at 300 K, but this is only true at Fermi level energy ($\mu -\mu_{F}=0$), or around Fermi level energy ($\mu -\mu_{F}\approx 0$), where *σ* also has its minimum values (almost zero). However, *S* could have its maximum values at 800 K instead of 300 K when the chemical potential *μ* has values that are not very close to *μ*_F_ value, and this is the main reason why PF has its maximum values at 800 K for most crystals at those values of chemical potential (*μ* has values that are not close to *μ*_F_). If the maximum PF occurs when *μ* − *μ*_F_ < 0 (hole doping region), then the implication is -that this material works better as a p-type TE material. Conversely, when *μ* − *μ*_F_ > 0 (electron doping region), then the crystal works better as an n-type TE material. The material should possess a high *σ* to present a high TE efficiency. The variation of *σ* with the chemical potential at three different temperatures is depicted in Figure S5. From the graph of electrical conductivity (*σ*/τ) in Figure S5, most crystals that have a high *σ*/τ in the hole doping region also have high PF values in this region compared to the electron doping region. Conversely, most crystals, which have a high *σ*/τ in the electron doping region, also have a high PF in this region compared to the hole doping region. The maximum *σ* for some crystals is observed for n-type doping, while it is observed for p-type in other crystals. One should keep in mind that these calculations were performed without fixing the carrier concentration. In order to know more about the temperature-dependent TE properties of these crystals, it is necessary to fix the carrier’s concentration. Figure 1 displays the figure of merit *ZT* with varying ranges of *μ* for the crystals: 1–4, 8–12, and 15–16 at three different temperatures (300 K, 500 K, and 800 K). Figure S7 in the SI shows *ZT* versus the chemical potential for the remaining 19 crystals. The highest values of *ZT* as a function of *μ* for these 30 crystals are represented in the sixth column of Table S2. From Table S2, ZT for 1-Tl_2_CdGeSe_4_, 2-Tl_2_HgSiSe_4_, 5-CuBS_2_, 6-CuBSe_2_, 15-AgAlSe_2_, 16-AgAlTe_2_, 20-AgGaS_2_, 29-NaInSe_2_, and 30-NaInTe_2_ is larger than unity.

The Seebeck coefficient predicted by the theory for metals and degenerate semiconductors, with a parabolic band, and the energy-independent charge carrier scattering approximation [107], depends on the carrier concentration of electrons or holes *n* and the effective mass m*. It can be given by [108]:

(7) $$S=\;\frac{8\pi^{2}k_{B}^{2}}{3eh^{2}}m^{*}\times T{\left(\frac{\pi }{3n}\right)}^{2/3}$$

where *n* is the carrier concentration (electrons or holes), *k_B_* and *e* are the Boltzmann constant and electronic charge, respectively. *S* is proportional to *T*, so *S* increases due to the increase in temperature. However, *κ_ele_* will also increase with increasing temperature, leading to reduced *ZT* and TE performance. Decreasing *ZT* with increasing *T* may be a problem for the TE performance, but it can be solved sometimes by changing the carrier concentration, which in turn will modify the Fermi level energy, leading to increased *ZT* with the increase in temperature. Doping (i.e., the introduction of either additional hole or electron carriers) has an enormous effect on electronic transport properties. Doping will set the Fermi level (*μ*_F_) and will directly influence the values of the transport properties.

In order to ensure that the Seebeck coefficient is large, there should only be a single type of carrier. Mixed n-type and p-type conduction will lead to both charge carriers moving to the cold end, canceling out the induced Seebeck voltages. Low carrier concentration insulators and even semiconductors may have large Seebeck coefficients; see Equation (6). However, low carrier concentration also results in low electrical conductivity. A compromise between large S and high electrical conductivity in thermoelectric materials must be struck to maximize the figure of merit *ZT (*$S^{2}\sigma T/\kappa$) where *κ* is the thermal conductivity.

This peak typically occurs at carrier concentrations between 10^18^ and 10^21^ carriers per cm^3^ (depending on the material system), which falls in between common metals and semiconductors—that is, concentrations found in heavily doped semiconductors. The next dataset describes the transport properties for both n-type and p-type systems at fixed doping levels ranging from 10^18^ to 10^21^ cm^−3^, increasing the doping by one order of magnitude at each step.

**Figure 2:**
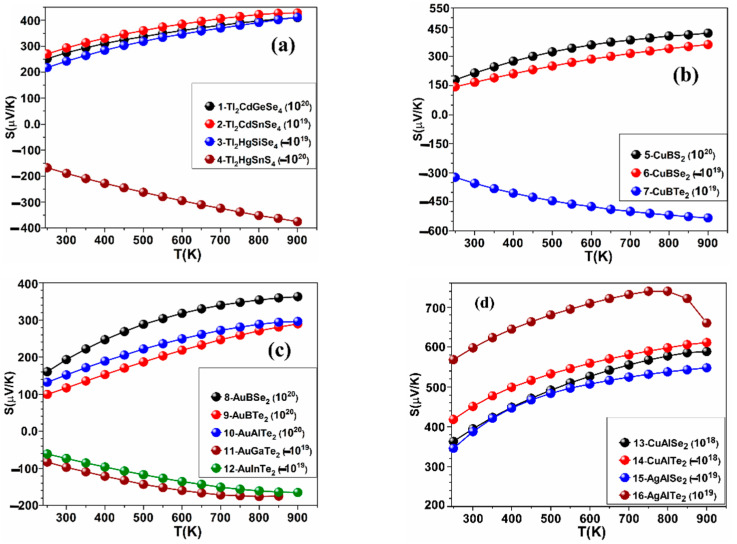
Calculated *S* versus temperature for (**a**) the crystals 1–4, (**b**) the crystals 5–7, (**c**) the crystals 8–12, and (**d**) the crystals 13–16.

**Figure 3:**
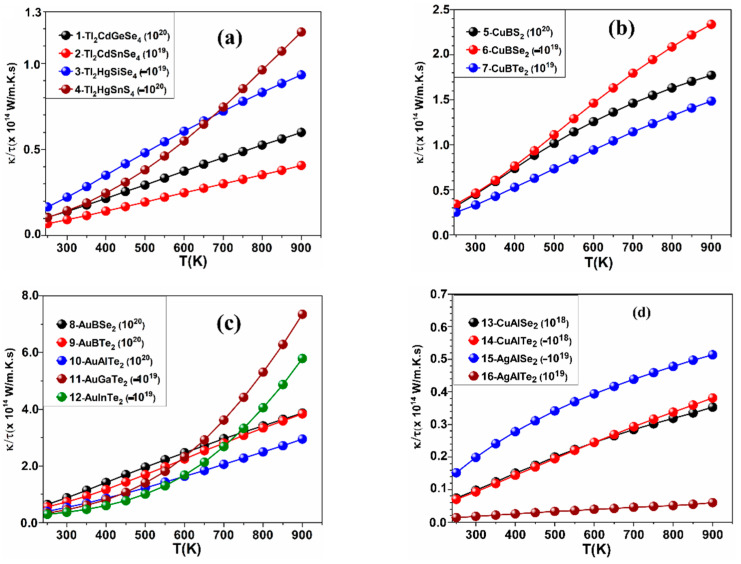
Calculated *κele*/τ the temperature for (**a**) the crystals 1–4, (**b**) the crystals 5–7, (**c**) the crystals 8–12, and (**d**) the crystals 13–16.

**Figure 4:**
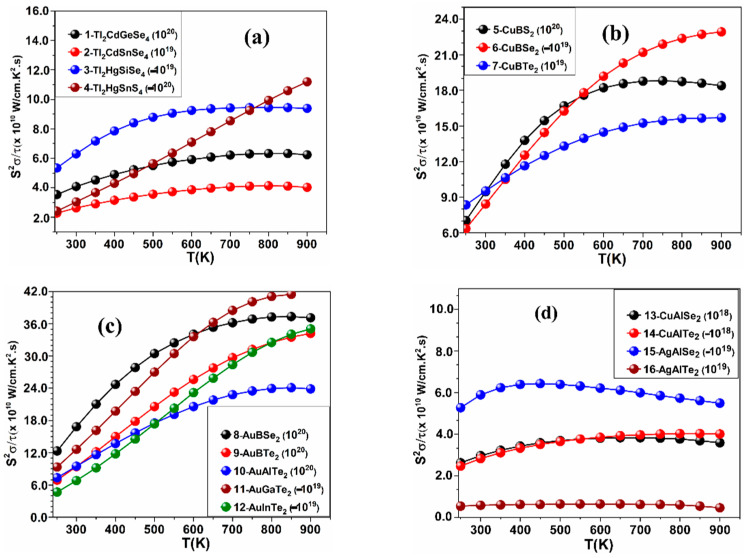
Calculated PF versus the temperature for (**a**) the crystals 1–4, (**b**) the crystals 5–7, (**c**) the crystals 8–12, and (**d**) the crystals 13–16.

**Figure 5:**
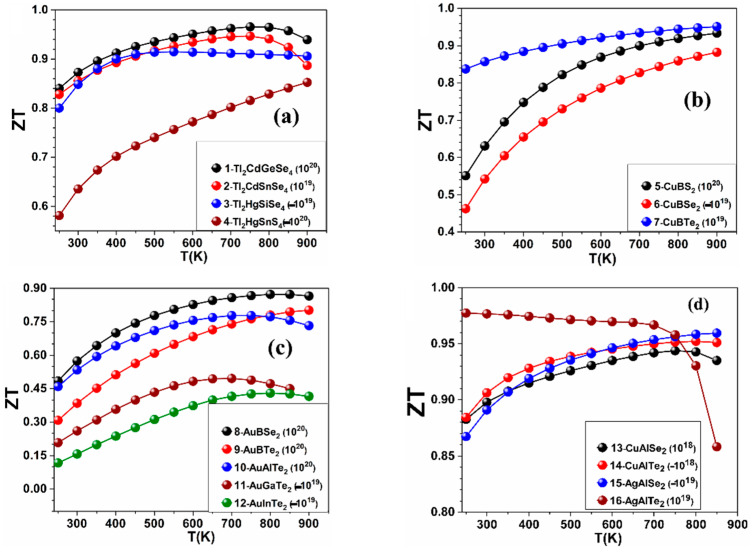
Calculated *ZT* versus the temperature for (**a**) the crystals 1–4, (**b**) the crystals 5–7, (**c**) the crystals 8–12, and (**d**) the crystals 13–16.

The calculated values of *S*, *κ_ele_*, PF, and *ZT* as a function of temperature at fixed values of carrier concentration in the ranges (±10^18^, ±10^19^, ±10^20^, ±10^21^ in e^−^/cm^3^) for the crystals: 1–16 are shown in Figure 2, Figure 3, Figure 4 and Figure 5, while the results for the remaining crystals are shown in Figures S9 and S10. A positive value of *n* indicates hole doping, while a negative value of *n* indicates electron doping. Figure 2 is for *S*, Figure 3 is for *κ_ele_*, Figure 4 is for the PF, and Figure 5 is for *ZT*. The temperature range was set to be from 250 K to 900 K. Table 1 shows the highest values of *ZT* for these crystals, the carrier concentrations for which the highest *ZT* values occur, the highest PFs, and the highest *κ_ele_*. In Figure 2, Figure 3, Figure 4 and Figure 5 and Table 1, the value of the carrier concentration (*n*) for each crystal was considered to be the value that can achieve the highest *ZT*. It is known that a narrow band gap correlates with a large carrier concentration because many charge carriers will transfer from the valence band to the conduction band. Figure 2 shows that *S* increases with increasing temperature at the indicated values of *n* and that *S* has positive values indicating p-type thermoelectric materials, except for the crystals 4-Tl_2_HgSnS_4_, 7-CuBTe_2_, 11-AuGaTe_2_, and 12-AuInTe_2_ for which *S* has negative values, indicating n-type TE materials. The crystal 8-AuBSe_2_ has the highest values of *S* and *ZT* among the five crystals: 8-AuBSe_2_, 9-AuBTe_2_, 10-AuAlTe_2_, 11-AuGaTe_2_, and 12-AuInTe_2_. As can be seen from Table 1 and Figure 2, Figure 3, Figure 4 and Figure 5, for most of these crystals, the highest values of *S* and *ZT* occur with hole doping, except for the crystals 3-Tl_2_HgSiSe_4_, 6-CuBSe_2_, 14-CuAlTe_2_, and 20-AgGaS_2_ where *S* and *ZT* can have the highest values with electron doping.

The values of the electronic thermal conductivity *κ_ele_*/τ and PF in Figure 3 and Figure 4 must be multiplied by the scattering time factor, which was taken to be 10^−14^ s in our study, as mentioned in Section 2 (computational methods). From Table 1, the highest *κ_ele_* occurs at 900 K for all 30 crystals. *κ_ele_* has the largest value (*κ_ele_* = 8.13 W/m.K) in the crystal 17-CuGaS2, while it has the smallest value (*κ_ele_* = 0.199 W/m.K) in the crystal 4-Tl_2_HgSnS_4_. Hence, the values of *κ_ele_* for all these 30 crystals are in the range from 0.199 W/m.K to 8.13 W/m.K, which indicates that the 30 crystals are very good TE materials if we know that the thermal conductivity value of the non-doped regular semiconductors such as silicon and germanium are 150 and 58 W/m.K for these two semiconductors respectively. Excellent TE materials should have a high PF (more than 3.0 mW/cm.K^2^). In general, if PF is greater than *κ* results in larger *ZT* and vice versa, namely 17-crystal CuGaS_2_ and 29-NaInSe2, as shown in Table 1. Some crystals, such as 4-Tl_2_HgSnS_2_, 18-CuGaSe_2_, 19-CuGaTe_2_, and 29-NaInSe_2,_ can have a high value of *ZT* at both regions (hole and electron doping). From Table 1, we notice an interesting fact about crystals: 18-CuGaSe_2_ and 23-CuInS_2_ can have a high value of *ZT* (0.784 and 0.81 respectively) at a low temperature (350 K) at the following values of *n*: −10^19^ and 10^20^ e^−^/cm^3^ respectively. Additionally, the crystals 19-CuGaTe_2_ and 26-AgInS_2_ can have high *ZT* (0.885 and 0.945, respectively) at 300 K at the values of *n*: −10^18^ and 10^18^ e^−^/cm^3^, respectively, and these are new and interesting findings.

The chemical potential-dependent transport properties of the crystals: 19-CuGaTe_2_, 22-AgGaTe_2_, 25-CuInTe_2_, and 28-AgInTe_2_ were calculated and can be found in the Supplementary Materials. Here, we focus only on the calculated *S* and *ZT* versus the temperature for these crystals at two wide ranges of carrier concentrations to reveal which one leads to a higher *S* and *ZT*. The ranges are: (+2 × 10^19^, +8 × 10^19^ in e^−^/cm^3^) and (+2 × 10^18^, +8 × 10^18^ in e^−^/cm^3^). Figure S11a–d shows *S* versus temperature for the crystals 19-CuGaTe_2_ and 22-AgGaTe_2_ over the two different ranges of *n*. *S* can reach its maximum value for the crystal 22-AgGaTe_2_ (*S* ≈ 310 μV/K) and for 19-CuGaTe_2_ (*S* ≈ 320 μV/K) at 650 K when *n* = 2 × 10^19^ for both these two crystals. In contrast, *S* can reach its maximum value at 450 K for 22-AgGaTe_2_ (*S* ≈ 325 μV/K) and at 400 K for 19-CuGaTe_2_ (*S* ≈ 400 μV/K) when *n* = 2 × 10^18^ for both crystals. Figure S11e–h shows our results for *ZT* at these two different ranges of *n*. For the crystals 19-CuGaTe_2_ and 22-AgGaTe_2_, in the first range, *ZT* hits its maximum value (*ZT* ≈ 0.8) at 500 K for the crystal 22-AgGaTe2 and at 450 K (*ZT* ≈ 0.82) for the crystal 19-CuGaTe_2_ when *n* = 2 × 10^19^ for both these two crystals. While when *n* = 8 × 10^19^, *ZT* has its highest value at 900 K for both crystals. For the same crystals, but in the second range of carrier concentrations, *ZT* reaches its maximum values (*ZT* ≈ 0.86 for 22-AgGaTe_2_, *ZT* ≈ 0.88 for 19-CuGaTe_2_) at 350 K when *n* = 2 × 10^18^ for both crystals. For the crystals 25-CuInTe_2_ and 28-AgInTe_2_ at the first range of *n*, the situation is a bit different. *ZT* versus *T* for these two crystals is shown in Figure S12. *ZT* for 28-AgInTe_2_ starts from its minimum value at 250 K then increases with increasing temperature to reach a maximum value (*ZT* ≈ 0.4) at 700 K when *n* = 3 × 10^19,^ then it starts decreasing. However, at 250 K, when *n* = 5 × 10^19^ *ZT* starts from its maximum value (*ZT* ≈ 0.84), it starts decreasing rapidly with increasing temperature. *ZT* for 25-CuInTe_2_ starts from its minimum value at 250 K then increases with increasing temperature to reach maximum value (*ZT* ≈ 0.4) at 600 K when *n* = 4 × 10^19^ then starts decreasing, while *ZT* starts from its maximum value (*ZT* ≈ 0.84) at 250 K when *n* = 6 × 10^19^ then starts decreasing rapidly with increasing temperature. These are totally new findings that have not been revealed yet in any previous work.

It is not easy to find previous experimental studies on the thermoelectric properties of these 30 crystals. Nevertheless, we have identified the following available experimental data. A study by Gui etc. [109] showed that *ZT* values at 850 K are 1.1, 0.87, and 1.0 for CuInS_2_, CuInSe_2_, and CuInTe_2_ crystals, respectively. Furthermore, our calculated *ZT* at 850 K was 0.65, 0.6, and 0.5 for these three crystals, respectively. Another four experimental studies [45,110,111,112] were conducted on the thermoelectric properties of CuInTe_2_ crystal. The first two studies showed that *ZT* ≈ 0.6 at 800 K, which is very close to ours (*ZT* = 0.56 at 800 K). The third and fourth studies showed that *ZT* ≈ 0.35 and *ZT* ≈ 0.4 at 700 K, which is less than ours (*ZT* = 0.69 at 700 K). Another two experimental studies [113,114] were carried out on the thermoelectric properties of CuGaTe_2_ crystal. Ref. [113] reported *ZT* ≈ 1.12 at 900 K, which is higher than our results (*ZT* = 0.67 at 900 K), while Ref. [114] showed that *ZT* ≈ 0.9 at 850 K, which is comparable to ours (*ZT* ≈ 0.7 at 850 K). On the other hand, Cao et al. and Yusufu et al. [58,115] experimentally found that *ZT* for AgGaTe_2_ to be about 0.5 at 800 K and 0.4 at 850 K, less than ours (*ZT* = 0.729 at 800, ZT = 0.724 at 850).

### 3.2. Transport Properties of AgSbSe_2_ and AgSbTe_2_ Crystals and the Doped Ones: AgSb_0.94_Cd_0.06_Te_2_ and AgSbTe_1.85_Se_0.15_

**Figure 6:**
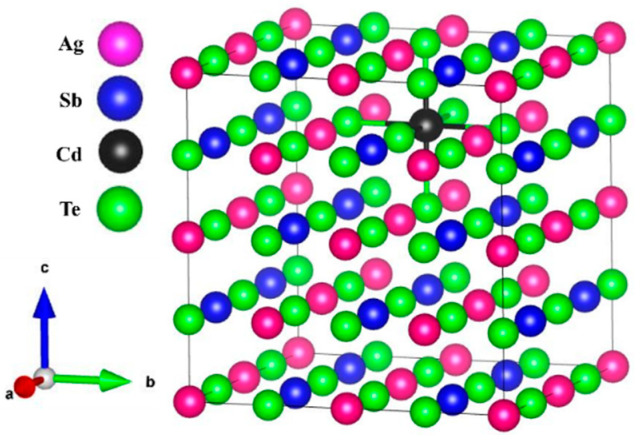
The crystal structure of AgSb_0.94_Cd_0.06_Te_2_ crystal.

**Figure 7:**
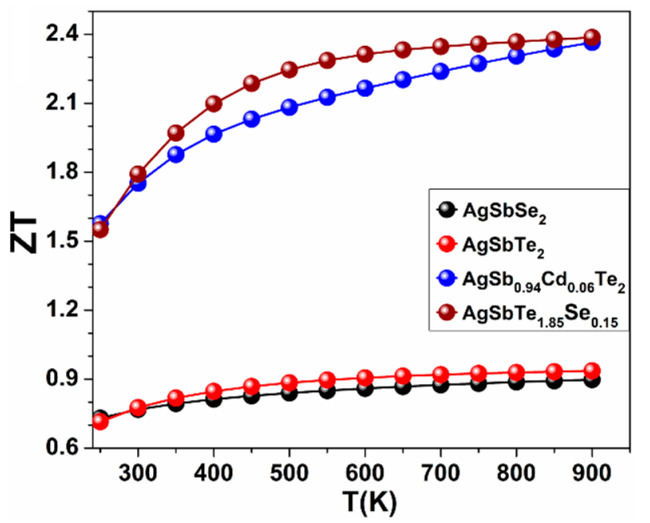
Calculated *ZT* versus the temperature for the pure AgSbSe_2_ and AgSbTe_2_ crystals, and the doped ones: AgSb_0.94_Cd_0.06_Te_2_ and AgSbTe_1.85_Se_0.15_.

**Figure 8:**
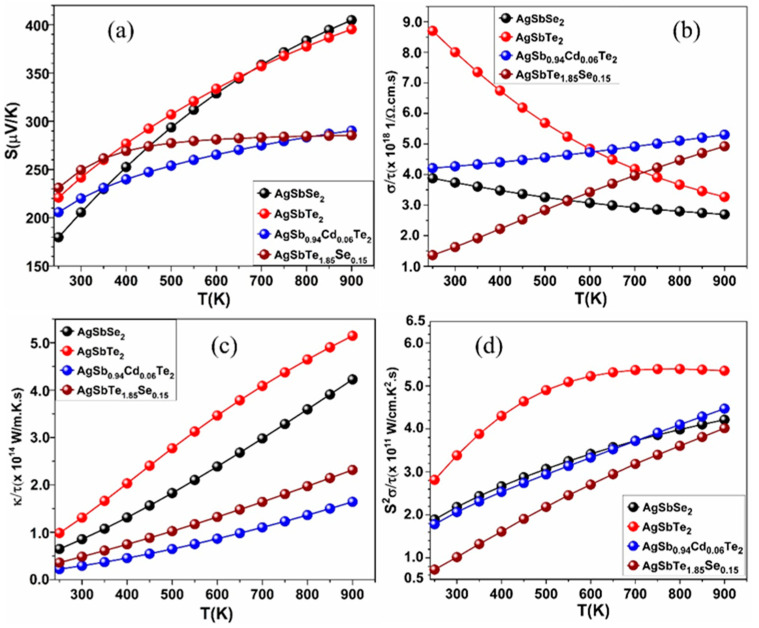
Calculated *S*, *σ*, *κ_ele_*, and PF versus the temperature for the pure AgSbSe_2_ and AgSbTe_2_ crystals, and the doped ones: AgSb_0__.94_Cd_0__.06_Te_2_ and AgSbTe_1__.85_Se_0.15_. (**a**) is for *S*, (**b**) is for *σ*, (**c**) is for *κ_ele_*, and (**d**) is for PF.

Cd-doped and Se-doped crystals (AgSb_0.94_Cd_0.06_Te_2_ and AgSbTe_1.85_Se_0.15_) arise from the substitution of Cd and Se atoms in AgSbTe_2_ crystal (Figure 6). The dopant compositions of AgSb_0.94_Cd_0.06_Te_2_ and AgSbTe_1.85_Se_0.15_ were chosen carefully based on very recent experimental study [46]. Our calculations for the band structure of AgSbSe_2_ and AgSbTe_2_ crystals showed that these crystals are semi-metallic materials. Our calculations for *ZT* are shown in Table 2, while Figure 7 and Figure 8 show our calculations for *ZT, S*, *σ*, *κ_ele_*, and PF as a function of temperature for the pure AgSbSe_2_ and AgSbTe_2_ crystals and for the doped ones (AgSb_0.94_Cd_0.06_Te_2_ and AgSbTe_1.85_Se_0.15_).

As can be seen from these figures, AgSb_0.94_Cd_0.06_Te_2_ and AgSbTe_1.85_Se_0.15_ crystals have less *S* and higher *σ* than the pure ones. Experimentally, *S* Roychowdhury et al. [46] showed that the hole concentration at 300 K of the pure AgSbTe_2_ increased from $4.3\times 10^{19}\;{\mathrm{cm}}^{-3}$ to $8.3\times 10^{19}\;{\mathrm{cm}}^{-3}$ when doped with 6 mol% Cd(AgSb_0.94_Cd_0.06_Te_2_), and that was the reason for reducing *S* values (*S* = ∝ 1/*n*) and increasing values of *σ* (σ ∝ *n*). Our study showed the same behavior for *S*. The doped crystals have less *κ_ele_* than the pure ones at high temperatures, and this makes the doped crystals have higher *ZT* than the pure crystals. This implies that doping by Cd and Se atoms results in reduced thermal conductivity and increases the TE performance. In addition, we contend that increasing the carrier concentration (*n*) does not necessarily mean an increase in the values of *κ_ele_* for the doped crystals. This can be true both experimentally and computationally since, in some cases, the doping process can create point defects, vacancies, and distortion in the bonding between atoms. This may lead to the reduction in the heat energy transferred by the charge carriers (electrons) or the phonons in the doped crystals.

Thermal conductivity has two components: lattice (phonons) and charge carriers. Increasing carrier concentration will increase thermal conductivity (if lattice does not change). However, for the dependence on temperature (our case), things may become more complicated. According to our DFT calculations, the crystals AgSbSe_2_ and AgSbTe_2_ are metallic materials with zero energy band gap. Wiedemann-Franz law basically relates the two conductivities of metals, i.e., thermal (*κ_ele_*) and electrical (*σ*) conductivity with temperature. It states that the ratio of thermal conductivity *κ_ele_* and electrical conductivity *σ* is proportional to the temperature of the specimen. G. Wiedemann and R. Franz established (based on experimental data) that the ratio $\frac{\kappa_{ele}}{\sigma }$ is constant at a constant temperature. L. Lorenz demonstrated that the relation $\frac{\kappa_{ele}}{\sigma }$ changes in direct proportion to the absolute temperature *T*, $\frac{\kappa_{ele}}{\sigma }=LT$, where *T* = temperature, *L* = 2.54 × 10^−8^ WΩ/K^2^, Lorentz number (a constant). This law basically states that with an increase in temperature, the thermal conductivity of metals increases while the electrical conductivity decreases. These two properties of metals are dependent on the free electrons. An increase in temperature increases the average velocity of the free electrons leading to an increase in heat energy transfer. On the other hand, an increase in the velocity of electrons also increases the number of collisions of the free electrons with lattice ions and hence contributes to an increase in electrical resistivity or reduction in electrical conductivity (*σ*), and this what can be noticed in Figure 8b,c for the crystals AgSbSe_2_ and AgSbTe_2_. However, this law has certain limitations. The proportionality does not hold true for all ranges of temperature. It is only found valid for very high temperatures and very low temperatures.

The above argument can be valid for the metallic materials, but few experimental studies showed that AgSbSe_2_ and AgSbTe_2_ crystals are semiconductors with a very narrow energy band gap. When we have semiconductors, alloys, or other interesting structures, the issue is much more complex, and it is difficult to obtain a general dependence of the conductivities with the temperature. That means the relationship between the thermal and electrical conductivities in the case of semiconducting materials is very complicated and is different from one material to another. Many researchers have tried to find a common formula that correlates between the two types of conductivities, and this relationship is still under study till now.

**Figure 9:**
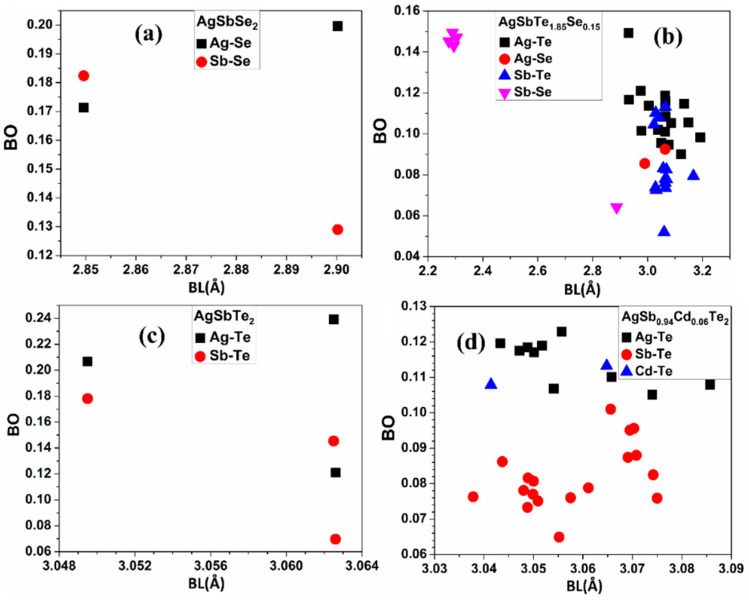
Calculated BO versus BL for the pure AgSbSe_2_ and AgSbTe_2_ crystals, and the doped ones: AgSb_0__.94_Cd_0__.06_Te_2_ and AgSbTe_1__.85_Se_0__.15_: (**a**) BO versus BL for AgSbSe_2_, (**b**) BO versus BL for AgSbTe_1.85_Se_0.15_, (**c**) BO versus BL for AgSbTe_2_, and (**d**) BO versus BL for AgSb_0__.94_Cd_0__.06_Te_2_.

The effective charge (Q*) on each atom of these four crystals is represented in Table 3, while Figure 9 shows the BO versus bond length (BL) for these four crystals. It is extremely helpful to investigate the interatomic bond strength between every pair of atoms in the crystal as represented by the BO values [92]. Important insights are revealed in Figure 9 and Table 3. In the AgSbSe_2_ crystal, there are two bonds: Ag-Se and Sb-Se, and these two bonds are ionic bonds (metal-nonmetal bonds) where the two metal elements (Ag, Sb) lose charge to the nonmetal chalcogen element (Se). While in the AgSbTe_2_ crystal, there are also two bonds: Ag-Te and Sb-Te, but the Ag-Te and Sb-Te bonds are more complicated to explain because Te is a metalloid element. Te sometimes acts similarly to a metal element and loses charge, and in different cases, it acts similarly to a nonmental element and gains charge (as our calculations show). As can be seen in Figure 9a,c, the Ag-Te bond is much stronger than the Ag-Se, Sb-Se, and Sb-Te bonds. The chalcogen elements (Se, Te) in each crystal of AgSbSe_2_ and AgSbTe_2_ make bonds with two atoms (two bond types): Ag and Sb. While each atom: Ag or Sb has just one bond type: Ag-Se and Sb-Se in AgSbSe_2_, and Ag-Te and Sb-Te in AgSbTe_2_. From the oxidation states of the Ag and Sb atoms, we know that each atom of these two atoms can bond with more than one or more different atoms in the crystal structure, and this leads to the fact that these two atoms can vibrate with higher frequency for phonons (higher thermal conductivity and less TE performance) than when they make two or three bonds with two or three other atoms in the crystal.

When the AgSbTe_2_ crystal is doped with Se atoms (AgSbTe_1.85_Se_0.15_), we notice from Figure 9b that each atom, Ag or Sb, now has two bond types and is bonded with two atoms (Se and Te) instead of just one atom, which we could not see in the pure AgSbTe_2_. Similarly, Figure 9d for the Te-doped crystal (AgSb_0.94_Cd_0.06_Te_2_) shows that the Te atom (heavy atom) is bonded now with three atoms (Ag, Sb, and Cd) instead of just two atoms (Ag and Sb) in the pure AgSbTe_2_. These three bonds in AgSb_0.94_Cd_0.06_Te_2_ make the heavy Te atom more bound and vibrate with lower frequencies for phonons. Hence, there is less thermal conductivity and higher TE performance. We conclude that the doping process of the AgSbTe_2_ crystal by Se and Cd dopants created new three bonds (Ag-Se, Sb-Se, and Cd-Te (Figure 8b–d) for atoms Ag, Sb, and Te and made them vibrate with lower frequencies for phonons (less thermal conductivity and higher TE performance) than in pure AgSbTe_2_, and this may explain why AgSbTe_1.85_Se_0.15_ and AgSb_0.94_Cd_0.06_Te_2_ crystals have less thermal conductivity and much higher ZT than the pure AgSbTe_2_ in terms of bonding properties.

Thermal conductivity in thermoelectric materials comes from two sources: (1) electrons and holes transporting heat (*κ_ele_*) and (2) phonons traveling through the lattice (*κ_lat_*). Most of the electronic term (*κ_ele_* is directly related to the electrical conductivity through the Wiedemann–Franz law:

(8) $$\kappa =\kappa_{ele}+\kappa_{lat}$$

(9) $$\kappa_{ele}=L\sigma T=LnemT$$

The Lorenz factor can vary, particularly with carrier concentration. Accurate assessment of *κ_ele_* is important, as *κ_lat_* is often computed as the difference between *κ* and *κ_ele_* (Equation (8)) using the experimental electrical conductivity. A common source of uncertainty in *κ_ele_* occurs in low-carrier-concentration materials where the Lorenz factor can be reduced by as much as 20% from the free-electron value. Additional uncertainty in *κ_ele_* arises from mixed conduction, which introduces a bipolar effect term into the thermal conductivity [117]. S Roychowdhury et al. [46] showed that the values of *κ_lat_* are in the range 0.5–0.55 W/m.K and in the range 0.1–0.13 W/m.K at the temperature range 300–575 K for the two crystals AgSbTe_2_ and AgSb_0.94_Cd_0.06_Te_2_ respectively. In contrast, our calculations showed that the values of *κ_ele_* are in the range 1.0–3.3 W/m.K and in the range 0.3–0.75 W/m.K at the temperature range 250–600 K for the two crystals AgSbTe2 and AgSb_0.94_Cd_0.06_Te_2_ respectively. This can be used as evidence that *κ_ele_* has a bigger effect on TE performance than *κ_lat_*. A further calculation of *κ_lat_* will help to explore why the doped crystals have much higher TE performance than the pure ones, which is not the focus of our study at this stage.

### 3.3. Correlation between Transport and Bonding Properties in 30 Chalcogenide Crystals

**Figure 10:**
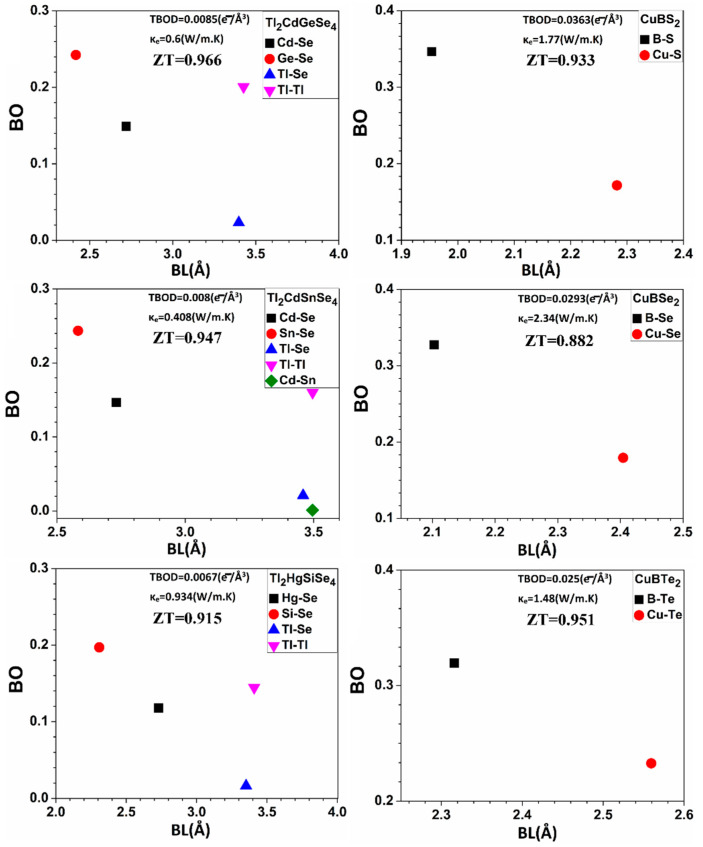
BO versus BL for the three crystals: (Tl_2_CdGeSe_4_, Tl_2_CdSnSe_4_, and Tl_2_HgSiSe_4_), and for the three crystals: (CuBS_2_, CuBSe_2_, and CuBTe_2_).

Identifying the underlying correlation between the TE performance (*ZT*) and the bonding characteristics of the 30 chalcogenide crystals is one of the main objectives of this work. In this regard, exploring the connection between the thermoelectric properties of the 30 crystals and the TBOD could be revealing. TBOD values for all 30 crystals are represented in Table S2 in the SI. TBOD describes the internal cohesion of the crystal, so that higher TBOD may imply a reduced value of thermal conductivity. In Figure S13, we plot the *ZT* versus TBOD for the 30 crystals. We notice that there is a general trend where *ZT* increases with the increase in TBOD. This correlation between TBOD and *ZT* could be revealed for the first time in this work. However, the crystals inside the blue circle shape (Tl_2_CdGeSe_4_, Tl_2_CdSnSe_4_, Tl_2_HgSiSe_4_, Tl_2_HgSnS_4_, NaInSe_2_, and NaInTe_2_) deviate from this trend. To explain this deviation, we plotted BO versus BL (Figure 10) for three crystals that deviate from the trend (Tl_2_CdGeSe_4_, Tl_2_CdSnSe_4_, and Tl_2_HgSiSe_4_) and for three crystals that follow the trend (CuBS_2_, CuBSe_2_, and CuBTe_2_). From Figure 10, Tl_2_CdGeSe_4_, Tl_2_CdSnSe_4_, and Tl_2_HgSiSe_4_ crystals have much lower TBOD values than CuBS_2_, CuBSe_2_, and CuBTe_2_ crystals, but they still have larger values of *ZT* than CuBS_2_, CuBSe_2_, and CuBTe_2_ crystals. It is true that Tl_2_CdGeSe_4_, Tl_2_CdSnSe_4_, and Tl_2_HgSiSe_4_ crystals have much lower TBOD values than CuBS_2_, CuBSe_2_, and CuBTe_2_ crystals, but at the same time, they have much smaller values of *κ_ele_* than CuBS_2_, CuBSe_2_, and CuBTe_2_ crystals, and this is the reason why Tl_2_CdGeSe_4_, Tl_2_CdSnSe_4_, and Tl_2_HgSiSe_4_ crystals still have higher values of *ZT* than CuBS_2_, CuBSe_2_, and CuBTe_2_ crystal (which have larger values of *κ_ele_*). The same explanation is valid for the other crystals (NaInSe_2_ and NaInTe_2_).

The crystal 19-CuGaTe_2_ has higher *ZT* (*ZT* = 0.662 at 900 K) than the crystal 18-CuGaSe_2_ (*ZT* = 0.556 at 900 K). The same trend can be seen in the crystals 27-AgInSe_2_ to 28-AgInTe_2_, where 28-AgInTe_2_ has *ZT* = 0.605 at 900 K while 27-AgInSe_2_ has *ZT* = 0.243 at the same temperature. In all these crystals, *ZT* decreases when moving from Te-related crystals toward Se-related crystals. In some crystals, this behavior can be related to the heavy Te atom, which results in less thermal conductivity and higher *ZT*. One of the important parameters that can affect the value of thermal conductivity is the bonding nature of the atoms in the crystal. Weaker bonds (lower bond order (BO)) imply higher energy phonon vibrations and, therefore, a higher value of *κ* and a reduced value of *ZT*. In contrast, stronger bonds (higher BO) imply smaller energy phonon vibrations and a reduced value of *κ*, and a higher value of *ZT*. Bonding properties calculations for these crystals have already been published in our previous work [79], and Table 4 represents our calculations for the bonding properties of the above crystals. As can be seen from Table 4, The crystals 19-CuGaTe_2_ and 28-AgInTe_2_ have bonds with higher bond order (stronger bonds) than the crystals 18-CuGaSe_2_ and 27-AgInSe_2_, and we believe that this is one of the main reasons that 19-CuGaTe_2_ and 28-AgInTe_2_ have smaller *κ* and higher *ZT* than the crystals: 18-CuGaSe_2_ and 27-AgInSe_2_.

## 4. Conclusions

A computational study was performed to investigate: 1. the thermoelectric transport properties of 30 chalcogenide crystals to provide a large set of thermoelectric transport data, which can be a good start to explore their further potentials both experimentally and theoretically, and 2. the temperature-dependent transport properties of the pure AgSbSe_2_ and AgSbTe_2_ and doped AgSb_0.94_Cd_0.06_Te_2_ and AgSbTe_1.85_Se_0.15_ crystals. In the first part (the 30 crystals), the transport properties of the crystals: 1-Tl2CdGeSe_4_, 2-Tl2CdSnSe_4_, 3-Tl_2_HgSiSe_4_, 4-Tl_2_HgSnS_4_, 8-AuBSe_2_, 9-AuBTe_2_, 10-AuAlTe_2_, 11-AuGaTe_2_, 12-AuInTe_2_, 15-AgAlSe_2_, and 16-AgAlTe_2_ are investigated for the first time. These eleven chalcogenide crystals are promising thermoelectric materials which have high Seebeck coefficients and a high figure of merit. For the crystals 19-CuGaTe_2_ and 22-AgGaTe_2_, we conclude that the second range of *n*: (+2 × 10^18^, +8 × 10^18^ in e^−^/cm^3^) leads to much better TE performance at low temperatures than the first range of *n*: (+2 × 10^19^, +8 × 10^19^ in e^−^/cm^3^). In the second part, it is found that the *ZT* value improves significantly, higher than 2.0 in p-type doped AgSb_0.94_Cd_0.06_Te_2_ and AgSbTe_1.85_Se_0.15_ crystals. Our calculations show a difference in the calculated values of the Seebeck coefficient and figure of merit for some crystals compared to other previous experimental and theoretical studies. Some correlations between the calculated bonding nature of these crystals and their thermoelectric properties were revealed in this work.

Despite a large number of simplified calculations on complex thermal transport properties, some for the first time, there are obviously some serious drawbacks, such as ignoring the lattice thermal conductivity. We are encouraged by the current results and aspire to continue research in this area for more complex and interesting chalcogenide crystals. It is desirable to improve the DFT calculations with better options, such as using either hybrid potential or Becke–Johnson potential. We should also point out that there are cases [65,118] where the neglect of SOC may not result in too large a difference due to unpredictable fluctuations. Our results without SOC are within the limit of these fluctuations and will still be useful as a first step to more accurate calculations. Enhancing the thermoelectric performance of these crystals can be achieved by decreasing the values of the thermal conductivity, which is our vision for future works. This could be performed by making these crystals as 2D and 1D materials.

## Figures and Tables

**Table 1:** The highest values of ZT and the values of the carrier concentrations (*n*) at which these values of ZT occur, highest PF, and highest *κ_ele_*/τ for the 30 chalcogenide crystals.

#	Crystal	*n* (e^−^/cm^3^)	Highest *ZT*	Highest PF (mW/cm.K^2^)	*κ_ele_* (W/m.K) at 900 K
1	Tl_2_CdGeSe_4_	10^20^	0.966(750 K)	0.634(800 K)	0.60
2	Tl_2_CdSnSe_4_	10^19^	0.947(750 K)	0.415(800 K)	0.408
3	Tl_2_HgSiSe_4_	−10^20^	0.915(550 K)	0.945(800 K)	0.934
4	Tl_2_HgSnS_4_	10^20^, −10^20^	0.943(900 K), 0.853(900 K)	1.49(850 K), 1.12(900 K)	0.199, 1.18
5	CuBS_2_	10^20^	0.933(900 K)	1.88(750 K)	1.77
6	CuBSe_2_	−10^19^	0.882(900 K)	2.29(900 K)	2.34
7	CuBTe_2_	10^19^	0.951(900 K)	1.57(850 K)	1.48
8	AuBSe_2_	10^20^, 10^19^	0.873(800 K), 0.889(650 K)	3.72(800 K), 1.57(650 K)	3.86, 1.7
9	AuBTe_2_	10^20^	0.802(900 K)	3.42(900 K)	3.84
10	AuAlTe_2_	10^20^	0.778(750 K)	2.35(750 K)	2.27
11	AuGaTe_2_	−10^20^, 10^20^	0.495(700 K), 0.55(400 K)	3.85(700 K), 1.58(400 K),	7.83, 5.87
12	AuInTe_2_	−10^19^, 10^20^	0.431(800 K), 0.536(400 K)	3.25(800 K)	7.57
13	CuAlSe_2_	10^18^, 10^19^, 10^20^	0.944(750 K), 0.935(850 K), 0.888(900 K)	0.38(750 K), 0.68(850 K), 2.15(900 K)	0.353, 0.647, 2.18
14	CuAlTe_2_	−10^18^, 10^18^	0.952(800 K), 0.947(850 K),	0.403(800 K), 0.577(850 K)	0.381, 0.549
15	AgAlSe_2_	−10^19^, −10^18^, 10^18^	0.960(900 K), 0.956(900 K), 0.955(900 K)	0.548(900 K), 0.748(900 K), 0.788(900 K)	0.514, 0.705, 0.743
16	AgAlTe_2_	10^20^, 10^19^	0.858(900 K), 0.977(250 K)	2.04(900 K)	2.14
17	CuGaS_2_	10^21^, 10^20^	0.607(900 K), 0.910(500 K)	5.48(900 K), 0.475(500 K)	8.13, 0.855
18	CuGaSe_2_	10^20^, −10^19^	0.688(600 K), 0.784(350 K)	2.26(600 K), 0.831(350 K)	3.77, 2.24
19	CuGaTe_2_	10^20^, −10^18^, 10^19^	0.739(700 K), 0.885(300 K), 0.847(400 K)	3.41(700 K), 0.352(300 K), 1.02(400 K)	4.96, 2.96, 3.04
20	AgGaS_2_	−10^20^, −10^19^	0.865(900 K), 0.823(900 K)	2.03(900 K), 2.81(900 K)	2.11, 3.07
21	AgGaSe_2_	10^20^	0.735(650 K)	1.86(650 K)	2.79
22	AgGaTe_2_	10^20^, 10^19^	0.729(800 K), 0.824(400 K)	2.86(900 K), 0.803(400 K)	3.76, 1.76
23	CuInS_2_	10^21^, 10^20^	0.633(900 K), 0.810(350 K)	5.03(900 K), 1.21(350 K)	7.16, 2.73
24	CuInSe_2_	10^21^	0.541(900 K)	4.67(900 K)	7.78
25	CuInTe_2_	10^20^	0.79(at 500 K)	1.85(500 K)	3.24
26	AgInS_2_	10^20^, 10^18^, −10^18^	0.824(750 K), 0.945(300 K), 0.986(250 K)	1.91(750 K), 0.151(300 K)	2.19, 0.75, 0.76
27	AgInSe_2_	10^20^	0.736(450 K)	1.31(450 K)	2.54
28	AgInTe_2_	10^20^	0.794(600 K)	1.97(600 K)	2.81
29	NaInSe_2_	−10^19^, −10^18^, 10^18^, 10^19^	0.956(900 K), 0.948(900 K), 0.946(900 K), 0.94(900 K)	0.308(900 K), 0.433(900 K), 0.457(900 K), 0.557(900 K)	0.29, 0.411, 0.435, 0.533
30	NaInTe_2_	10^20^	0.952(850 K)	0.335(850 K)	0.565

**Table 2:** Our calculated ZT at 900 K and a comparison with other experimental works for the pure AgSbSe_2_ and AgSbTe_2_ crystals, and the doped ones: AgSb_0_._94_Cd_0_._06_Te_2_ and AgSbTe_1.85_Se_0_._15_.

Crystal	*n*	ZT (Ours)	ZT
AgSbSe_2_	10^20^	0.898(750 K)	0.41(650 K) [71], 0.65(675 K) [116]
AgSbTe_2_	10^19^	0.924(750 K)	1.2(650 K) [47], 0.9(675 K) [76]
AgSb_0.94_Cd_0.06_Te_2_	9.0 × 10^19^	2.36(700 K)	2.6(573 K) [46]
AgSbTe_1.85_Se_0.15_	10^20^	2.39(700 K)	2.1(575 K) [21,47]

**Table 3:** Calculated average effective charge for pure AgSbSe_2_ and AgSbTe_2_ crystals, and the doped ones: AgSb_0.94_Cd_0.06_Te_2_ and AgSbTe_1.85_Se_0.15_.

#	Crystal	Q* (in e^−^)
1	AgSbSe_2_	10.971(Ag), 4.399(Sb), 6.315(Se)
2	AgSbTe_2_	11.102(Ag), 4.694(Sb), 6.102(Te)
3	AgSb_0.94_Cd_0.06_Te_2_	11.080(Ag), 4.748(Sb), 11.243(Cd), 6.102(Te)
4	AgSbTe_1.85_Se_0_._15_	11.030(Ag), 4.712(Sb), 6.120(Te), 6.266(Se)

**Table 4:** Bonding properties of 18-CuGaSe_2_, 19-CuGaTe_2_, 27-AgInSe_2_, and 28-AgInTe_2_ crystals.

CuGaSe_2_			CuGaTe_2_		
**bond**	**BL(Å)**	**BO**	**bond**	**BL(Å)**	**BO**
Cu-Se	2.4315	0.1916	Cu-Te	2.5937	0.2286
Ga-Se	2.4746	0.2572	Ga-Te	2.6878	0.2709
**AgInSe_2_**			**AgInTe_2_**		
**bond**	**BL(Å)**	**BO**	**bond**	**BL(Å)**	**BO**
Ag-Se	2.6727	0.1530	Ag-Te	2.8134	0.1814
In-Se	2.6558	0.2384	In-Te	2.8603	0.2581

## Data Availability

The data used to support the findings of this study are included within the article.

## Supplementary Materials

The following supporting information can be downloaded at: https://www.mdpi.com/article/10.3390/ma15082843/s1, Figure S1: Calculated Seebeck coefficient (S) versus the chemical potential for the crystals from 1-Tl_2_CdGeS_4_ to 16-AgAlTe_2_; Figure S2: Calculated PF versus the chemical potential for the crystals from 1-Tl_2_CdGeS4 to 16-AgAlTe_2_; Figure S3: Calculated Seebeck coefficient (S) versus the chemical potential for the crystals from 17-CuGaS_2_ to 30-NaInTe_2_; Figure S4: Calculated PF versus the chemical potential for the crystals from 17-CuGaS_2_ to 30-NaInTe_2_; Figure S5: Calculated σ/τ versus the chemical potential for the 30 crystals; Figure S6: Calculated κ/τ versus the chemical potential for the 30 crystals; Figure S7: Calculated ZT versus the chemical potential; Figure S8: Calculated σ/τ versus the temperature for the crystals 13–16; Figure S9: Calculated S and PF versus the temperature for the crystals from 17 to 30; Figure S10: Calculated ZT versus the temperature for the crystals from 17 to 30; Figure S11: S and ZT versus the temperature for the crystals: (a,b,e,f) are for 19-CuGaTe2 crystal, and (c,d,g,h) are for 22-AgGaTe2 crystal at two different ranges of *n*; Figure S12: Shows our results for ZT versus temperature at range of *n*: (+2 × 10^19^, +8 × 10^19^ in e-/cm^3^) for the crystals 25-CuInTe2 and 28-AgInTe2; Figure S13: Calculated TBOD versus ZT for the 30 crystals. Table S1: The fully optimized structures with the corresponding experimental lattice parameters of 30 chalcogenide crystals; Table S2: Energy gap (Eg) (calculated in our previous works), the Seebeck coefficients (S), and figure of merit (ZT) as a function of chemical potential at room temperature (300 K), and the TBOD for these 30 chalcogenide crystals. In the previous published works (Refs. [79,80] in the main text), OLCAO code was used to calculate the energy band gap. Spin–Orbit Coupling (SOC) effect was not included in the calculations [119,120,121,122].
