# Supplementary material for: First-Principles Calculations of Thermoelectric Transport Properties of Quaternary and Ternary Bulk Chalcogenide Crystals
Source: Materials (Basel). 2022 Apr 13;15(8):2843. doi: 10.3390/ma15082843 (PMC9032660; doi:10.3390/ma15082843)
Supplement: Supplementary file 1 [file materials-15-02843-s001.zip › materials-1623353-supplementary.pdf]

materials

Article

First-Principles Calculations of Thermoelectric Transport Properties of Quaternary and Ternary Bulk Chalcogenide Crystals

Sahib Hasan [1,2], Saro San [1], Khagendra Baral [1], Neng Li [3], Paul Rulis [1] and Wai-Yim Ching [1,*]

1 Department of Physics and Astronomy, University of Missouri-Kansas City, Kansas City, MO 64110, USA; sahmvd@mail.umkc.edu (S.H.); ssawcc@mail.umkc.edu (S.S.); kbx67@mail.umkc.edu (K.B.); rulisp@umkc.edu (P.R.)
2 Department of Sciences, College of Basic Education, Al Muthanna University, Samawah 66001, Iraq
3 School of Materials Science and Engineering, Wuhan University of Technology, No. 122, Luoshi Road, Wuhan 430070, China; lineng@whut.edu.cn
* Correspondence: chingw@umkc.edu

Citation: Hasan, S.; San, S.; Baral, K.; Li, N.; Rulis, P.; Ching, W.-Y. First-Principles Calculations of Thermoelectric Transport Properties of Quaternary and Ternary Bulk Chalcogenide Crystals. *Materials* **2022**, *15*, 2843. https://doi.org/10.3390/ma15082843

Academic Editors: Irena Jankowska-Sumara and Magdalena Krupska-Klimczak

Received: 18 February 2022
Accepted: 9 April 2022
Published: 13 April 2022

Publisher's Note: MDPI stays neutral with regard to jurisdictional claims in published maps and institutional affiliations.

Copyright: © 2022 by the authors. Submitted for possible open access publication under the terms and conditions of the Creative Commons Attribution (CC BY) license (https://creativecommons.org/licenses/by/4.0/).

1. The Supplementary Tables

Table S1. The fully optimized structures with the corresponding experimental lattice parameters of 30 chalcogenide crystals.

#	Crystal	Space group	Lattice parameters (our calculations) a, b, c(Å), α, β, γ(degree)	Lattice parameters(experimental) a, b, c(Å), α, β, γ(degree)
1	$Tl_2CdGeSe_4$	Tet, I-4 2 m(121)	8.1791, 8.1791, 6.8532, 90.00, 90.00, 90.00	8.0145, 8.0145, 6.7234, 90.00, 90.00, 90.00[1]
2	$Tl_2CdSnSe_4$	Tet, I-4 2 m(121)	8.2291, 8.2291, 6.9925, 90.00, 90.00, 90.00	8.0490, 8.0490, 6.8573, 90.00, 90.00, 90.00[1]
3	$Tl_2HgSiSe_4$	Tet, I-4 2 m(121)	8.1599, 8.1599, 6.8546, 90.00, 90.00, 90.00	8.0032, 8.0032, 6.6879, 90.00, 90.00, 90.00[2]
4	Tl_2HgSnS_4	Tet, I-4 2 m(121)	7.9589, 7.9589, 6.8578, 90.00, 90.00, 90.00	7.8571, 7.85716.6989, 90.00, 90.00, 90.00[3]
5	$CuBS_2$	Tet, I-42d(122)	5.0203, 5.0203, 9.0696, 90.00, 90.00, 90.00	5.044, 5.044, 8.948056, 90.00, 90.00, 90.00[4]
6	$CuBSe_2$	Tet, I-42d(122)	5.3328, 5.3328, 9.7364, 90.00, 90.00, 90.00	5.782, 5.782, 11.4021, 90.00, 90.00, 90.00[4]
7	$CuBTe_2$	Tet, I-42d(122)	5.7218, 5.7218, 10.7973, 90.00, 90.00, 90.00	5.616, 5.616, 10.6479, 90.00, 90.00, 90.00[4]
8	$AuBSe_2$	Tet, I-42d(122)	5.6726, 5.6726, 9.6556, 90.00, 90.00, 90.00	5.676, 5.676, 9.6776, 90.00, 90.00, 90.00[5]
9	$AuBTe_2$	Tet, I-42d(122)	5.9911, 5.9911, 10.8396, 90.00, 90.00, 90.00	6.011, 6.011, 10.7897, 90.00, 90.00, 90.00[5]
10	$AuAlTe_2$	Tet, I-42d(122)	6.3156, 6.3156, 12.3673, 90.00, 90.00, 90.00	6.244, 6.244, 12.5692, 90.00, 90.00, 90.00[5]
11	$AuGaTe_2$	Tet, I-42d(122)	6.3232, 6.3232, 12.4756, 90.00, 90.00, 90.00	6.2990, 6.2990, 12.4720, 90.00, 90.00, 90.00[5]
12	$AuInTe_2$	Tet, I-42d(122)	6.5064, 6.5064, 13.0550, 90.00, 90.00, 90.00	6.4840, 6.4840, 12.9745, 90.00, 90.00, 90.00[5]
13	$CuAlSe_2$	Tet, I-42d(122)	5.6526, 5.6526, 11.1557, 90.00, 90.00, 90.00	5.602, 5.602, 10.944, 90.00, 90.00, 90.00[6]
14	$CuAlTe_2$	Tet, I-42d(122)	6.0944, 6.0944, 12.1101, 90.00, 90.00, 90.00	5.964, 5.964, 11.780, 90.00, 90.00, 90.00[6]
15	$AgAlSe_2$	Tet, I-42d(122)	6.0324, 6.0324, 11.1150, 90.00, 90.00, 90.00	5.95, 5.95, 10.7457, 90.00, 90.00, 90.00[7]
16	$AgAlTe_2$	Tet, I-42d(122)	6.4134, 6.4134, 12.2311, 90.00, 90.00, 90.00	6.29, 6.29, 11.8252, 90.00, 90.00, 90.00[7]
17	$CuGaS_2$	Tet, I-42d(122)	5.3824, 5.3824, 10.6673, 90.00, 90.00, 90.00	5.347, 5.347, 10.974, 90.00, 90.00, 90.00[8]
17	$CuGaSe_2$	Tet, I-42d(122)	5.6782, 5.6782, 11.2749, 90.00, 90.00, 90.00	5.607, 5.607, 10.990, 90.00, 90.00, 90.00[8]
19	$CuGaTe_2$	Tet, I-42d(122)	6.0999, 6.0999, 12.1799, 90.00, 90.00, 90.00	5.994, 5.994, 11.912, 90.00, 90.00, 90.00[8]
20	$AgGaS_2$	Tet, I-42d(122)	5.7803, 5.7803, 10.6678, 90.00, 90.00, 90.00	5.755, 5.755, 10.283, 90.00, 90.00, 90.00[8]
21	$AgGaSe_2$	Tet, I-42d(122)	6.0507, 6.0507, 11.2857, 90.00, 90.00, 90.00	5.985, 5.985, 10.901, 90.00, 90.00, 90.00[8]
22	$AgGaTe_2$	Tet, I-42d(122)	6.4089, 6.4089, 12.3514, 90.00, 90.00, 90.00	6.301, 6.301, 11.962, 90.00, 90.00, 90.00[8]

23	$CuInS_2$	Tet, I-42d(122)	5.594, 5.594, 11.273, 90.00, 90.00, 90.00	5.522, 5.522, 11.132, 90.00, 90.00, 90.00[8]
24	$CuInSe_2$	Tet, I-42d(122)	5.879, 5.879, 11.829, 90.00, 90.00, 90.00	5.785, 5.785, 11.570, 90.00, 90.00, 90.00[8]
25	$CuInTe_2$	Tet, I-42d(122)	6.302, 6.302, 12.648, 90.00, 90.00, 90.00	6.179, 6.179, 12.360, 90.00, 90.00, 90.00[8]
26	$AgInS_2$	Tet, I-42d(122)	5.936, 5.936, 11.567, 90.00, 90.00, 90.00	5.827, 5.827, 11.190, 90.00, 90.00, 90.00[8]
27	$AgInSe_2$	Tet, I-42d(122)	6.197, 6.197, 12.123, 90.00, 90.00, 90.00	6.098, 6.098, 11.708, 90.00, 90.00, 90.00[8]
28	$AgInTe_2$	Tet, I-42d(122)	6.571, 6.571, 13.021, 90.00, 90.00, 90.00	6.443, 6.443, 12.636, 90.00, 90.00, 90.00[8]
29	$NaInSe_2$	Tet, I-42d(122)	6.7103, 6.7103, 11.2860, 90.00, 90.00, 90.00	6.418, 6.418, 12.644, 90.00, 90.00, 90.00[9]
30	$NaInTe_2$	Tet, I-42d(122)	7.1943, 7.1943, 12.2738, 90.00, 90.00, 90.00	6.599, 6.599, 12.276, 90.00, 90.00, 90.00[9]

Table S2. Energy gap (Eg) (calculated in our previous works), the Seebeck coefficients (S) and figure of merit (ZT) as a function of chemical potential at room temperature (300 K), and the TBOD for these 30 chalcogenide crystals. In the previous published works (Refs. 79 and 80 in the main text), OLCAO code was used to calculate the energy band gap. Spin-Orbit Coupling (SOC) effect was not included in the calculations.

#	Crystal	Eg(eV)	S(μV/K)	S(μV/K)	Highest ZT	TBOD (e^-/ Å 3)
1	$Tl_2CdGeSe_4$	0.814(D)	1300	—	1.01	0.0085
2	$Tl_2CdSnSe_4$	0.810(D)	1250	—	1.0	0.0080
3	$Tl_2HgSiSe_4$	0.992(D)	1500	—	1.02	0.0067
4	Tl_2HgSnS_4	1.105(D)	1660	—	1.0	0.0066
5	$CuBS_2$	1.8(D)	2600	2295[4]	1.01	0.0363
6	$CuBSe_2$	1.6(D)	2500	2527[4]	1.02	0.0293
7	$CuBTe_2$	1.2(D)	1800	1704[4]	1.0	0.0250
8	$AuBSe_2$	1.78(ID)	925	—	0.96	0.0228
9	$AuBTe_2$	1.40(ID)	825	—	0.98	0.0202
10	$AuAlTe_2$	1.60(D)	600	—	0.94	0.0151
11	$AuGaTe_2$	0.646(D)	205	—	0.60	0.0144
12	$AuInTe_2$	0.635(D)	190	—	0.54	0.0125
13	$CuAlSe_2$	1.20(D)	1600	—	1.0	0.0209
14	$CuAlTe_2$	1.70(ID)	1800	—	1.0	0.0182
15	$AgAlSe_2$	1.45(D)	2050	—	1.005	0.0170
16	$AgAlTe_2$	1.81(D)	1900	—	1.01	0.0149
17	$CuGaS_2$	0.80(D)	850	—	0.96	0.0234
18	$CuGaSe_2$	0.39(D)	700	—	0.82	0.0198
19	$CuGaTe_2$	0.85(D)	550	—	0.90	0.0176
20	$AgGaS_2$	1.05(D)	1100	—	1.01	0.0185
21	$AgGaSe_2$	0.60(D)	480	—	0.90	0.0160
22	$AgGaTe_2$	0.90(D)	500	860[10]	0.91	0.0144
23	$CuInS_2$	0.240(D)	390	370[11]	0.84	0.0191
24	$CuInSe_2$	0.146(D)	260	240[11]	0.70	0.0166
25	$CuInTe_2$	0.754(D)	460	325[11], 00[12]	0.88	0.0153
26	$AgInS_2$	0.651(D)	770	—	0.98	0.0152
27	$AgInSe_2$	0.453(D)	390	—	0.84	0.0135
28	$AgInTe_2$	1.001(D)	525	500[13], 750[14]	0.92	0.0125
29	$NaInSe_2$	1.54(D)	2400	—	1.01	0.0111
30	$NaInTe_2$	1.80(D)	2100	—	1.02	0.00942

2. The Supplementary Figures

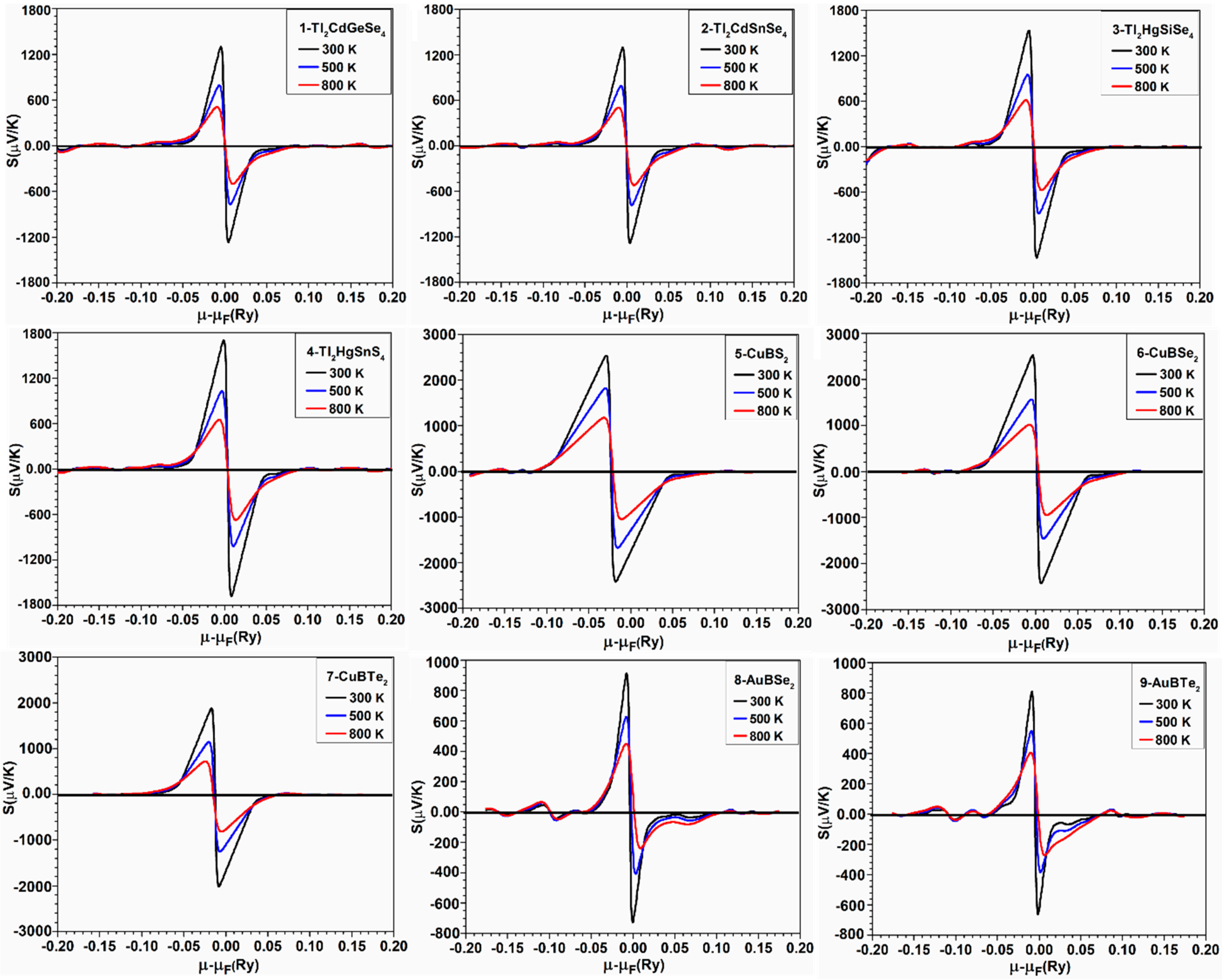

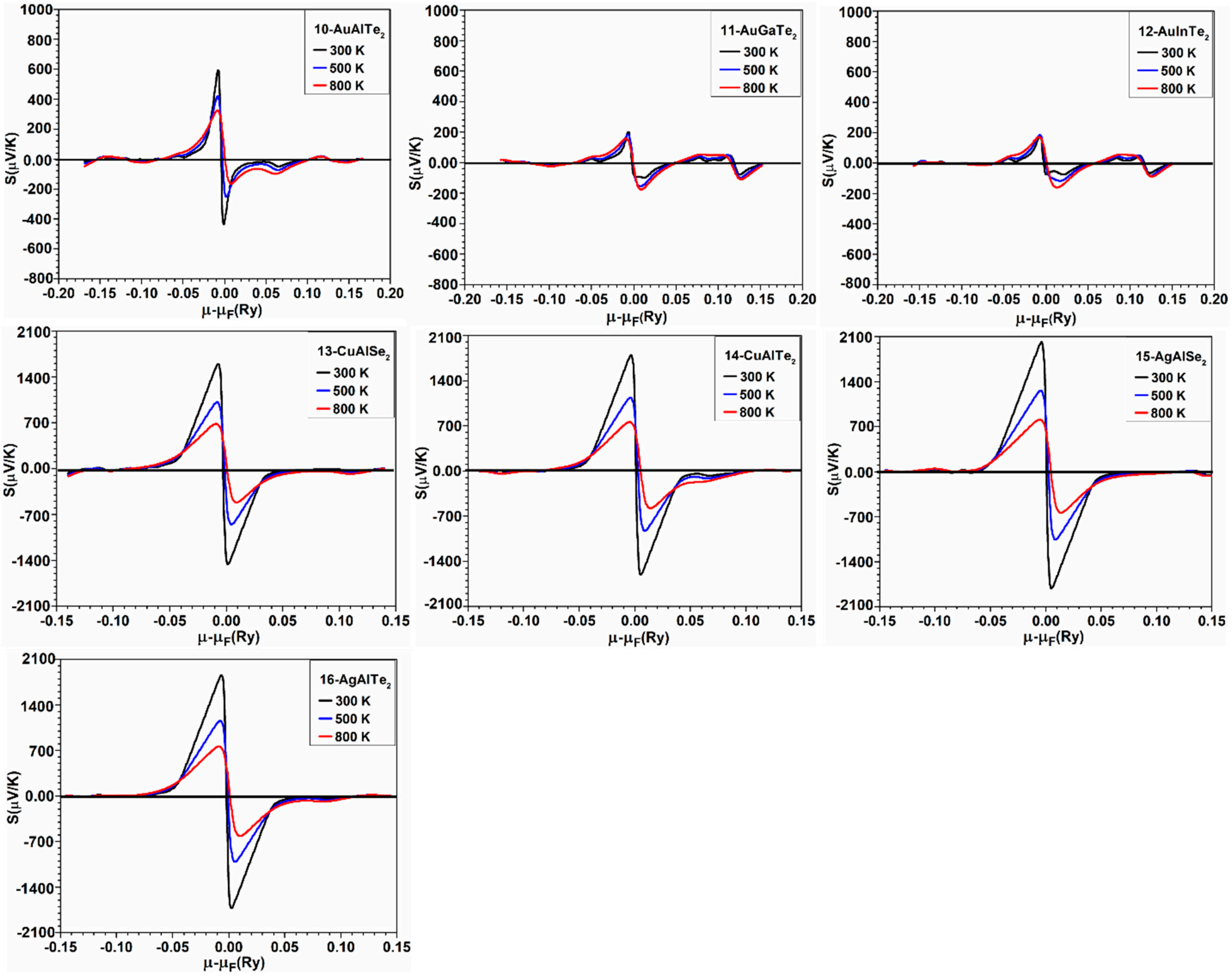

Figure S1. Calculated Seebeck coefficient(S) versus the chemical potential for the crystals from 1-Tl_2CdGeS_4 to 16-$AgAlTe_2$.

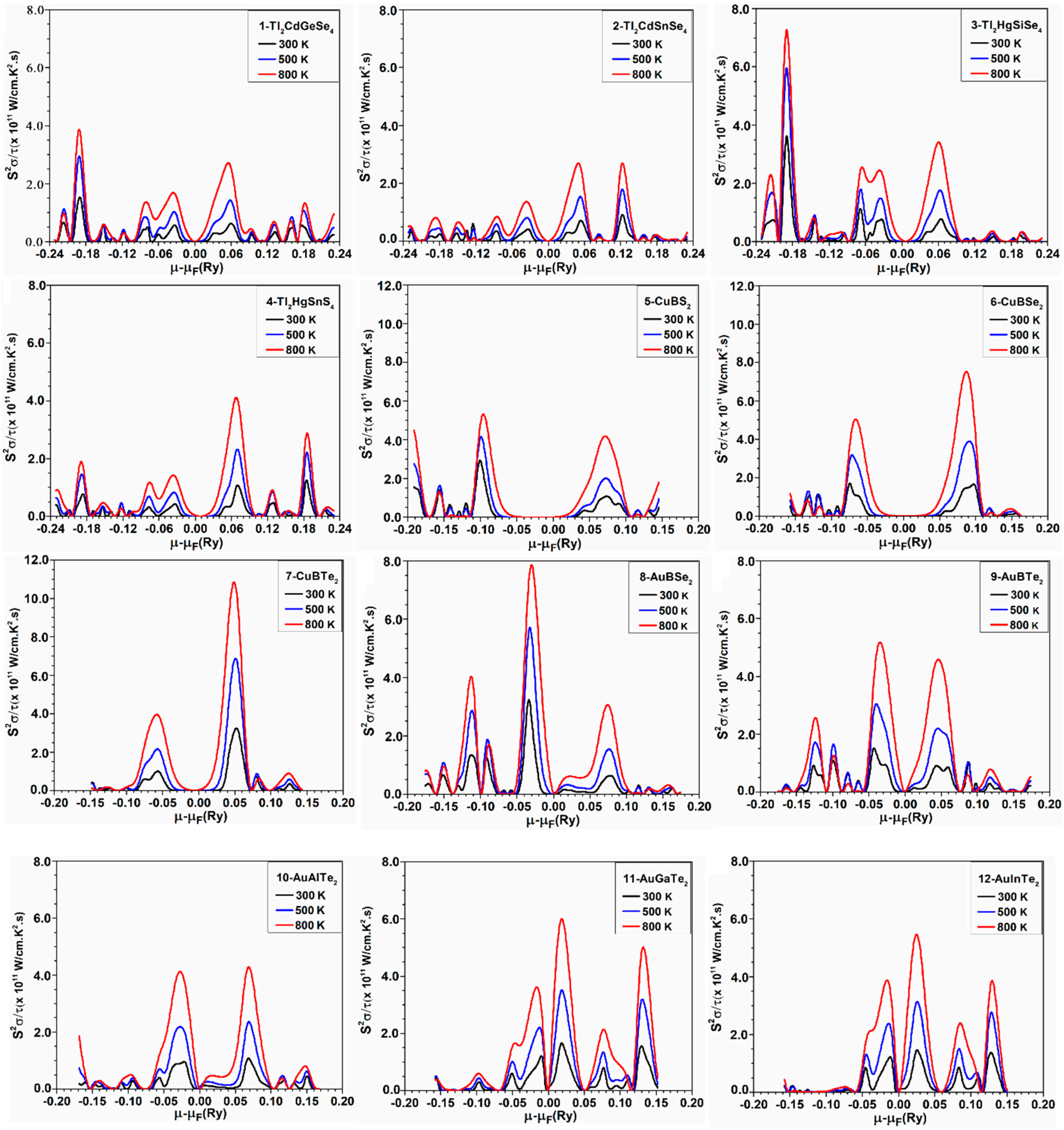
1-Tl$_2$CdGeSe$_4$
2-Tl$_2$CdSnSe$_4$
3-Tl$_2$HgSiSe$_4$
4-Tl$_2$HgSnS$_4$
5-CuBS$_2$
6-CuBSe$_2$
7-CuBTe$_2$
8-AuBSe$_2$
9-AuBTe$_2$
10-AuAlTe$_2$
11-AuGaTe$_2$
12-AuInTe$_2$
300 K
500 K
800 K
$S^2\sigma/\tau$(x 10^{11} W/cm.K^2.s)
$\mu-\mu_F$(Ry)

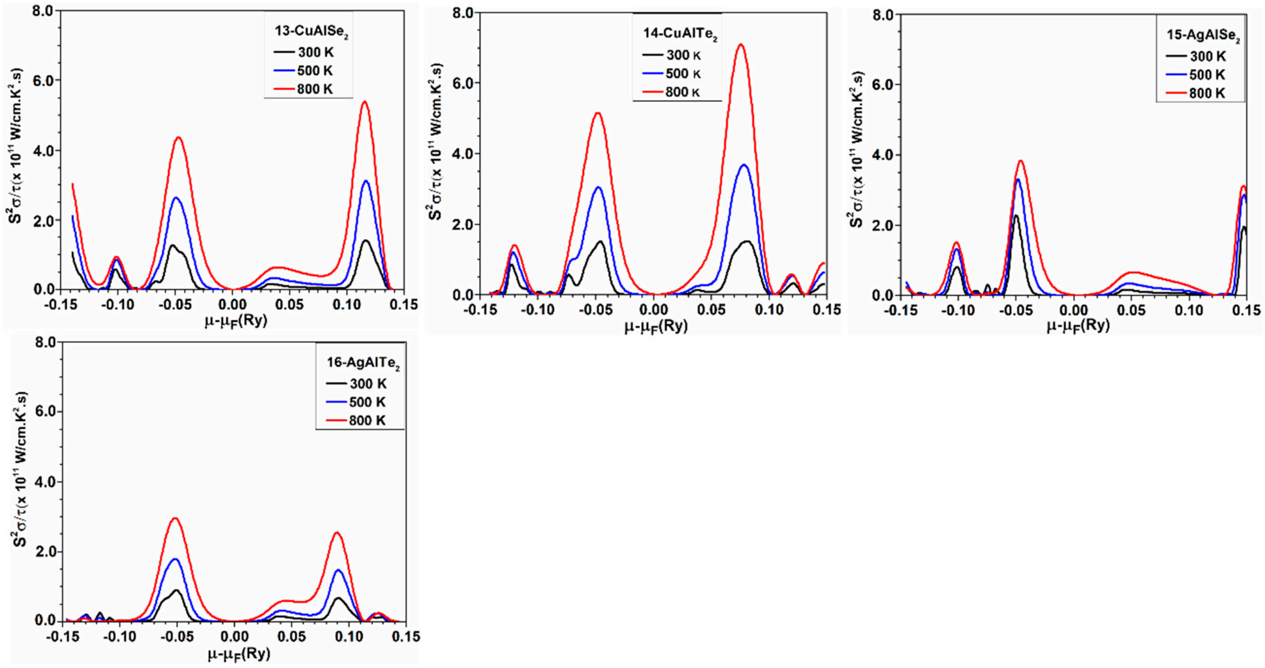

Figure S2. Calculated PF versus the chemical potential for the crystals from 1-$Tl_2CdGeS4$ to 16-$AgAlTe_2$.

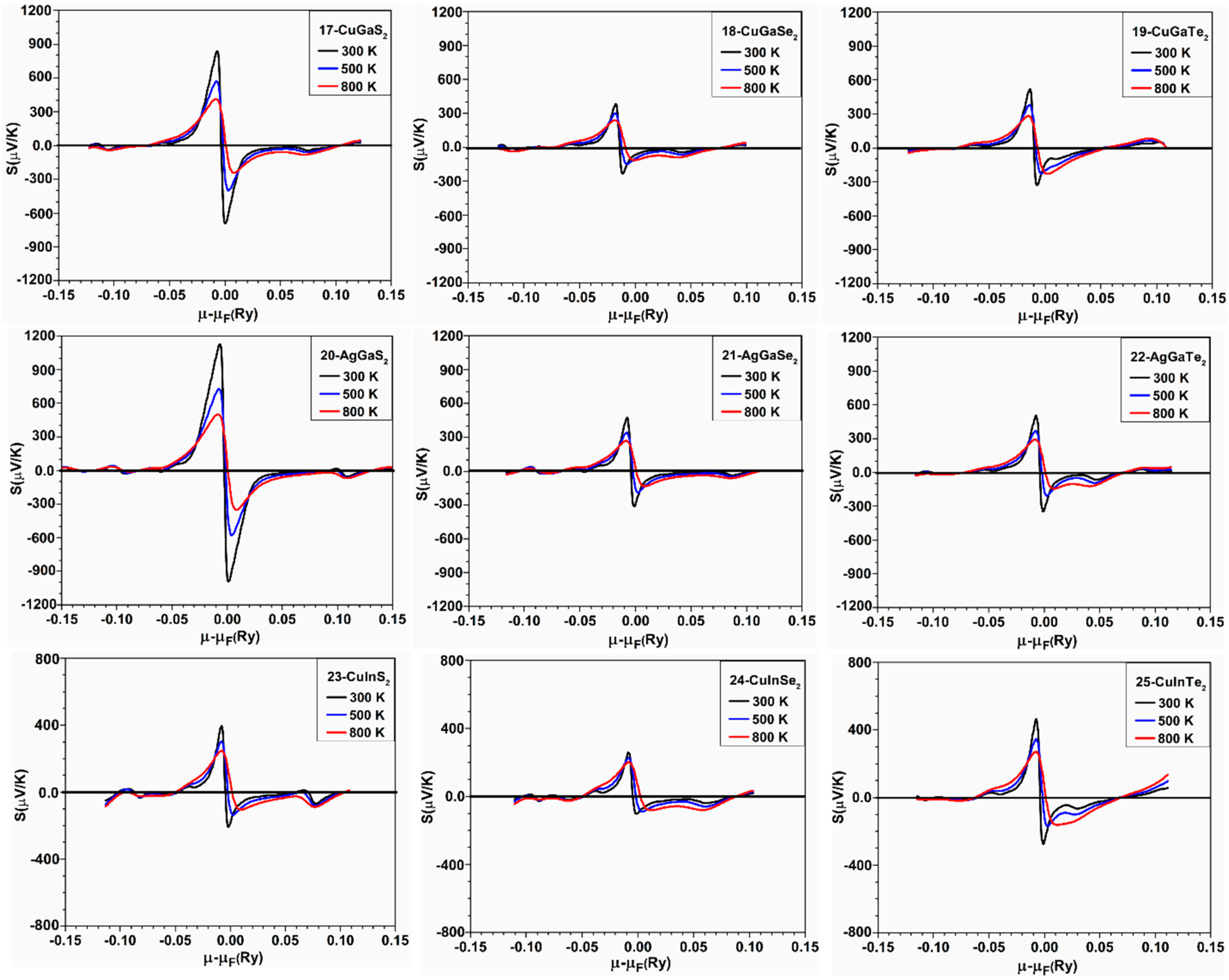

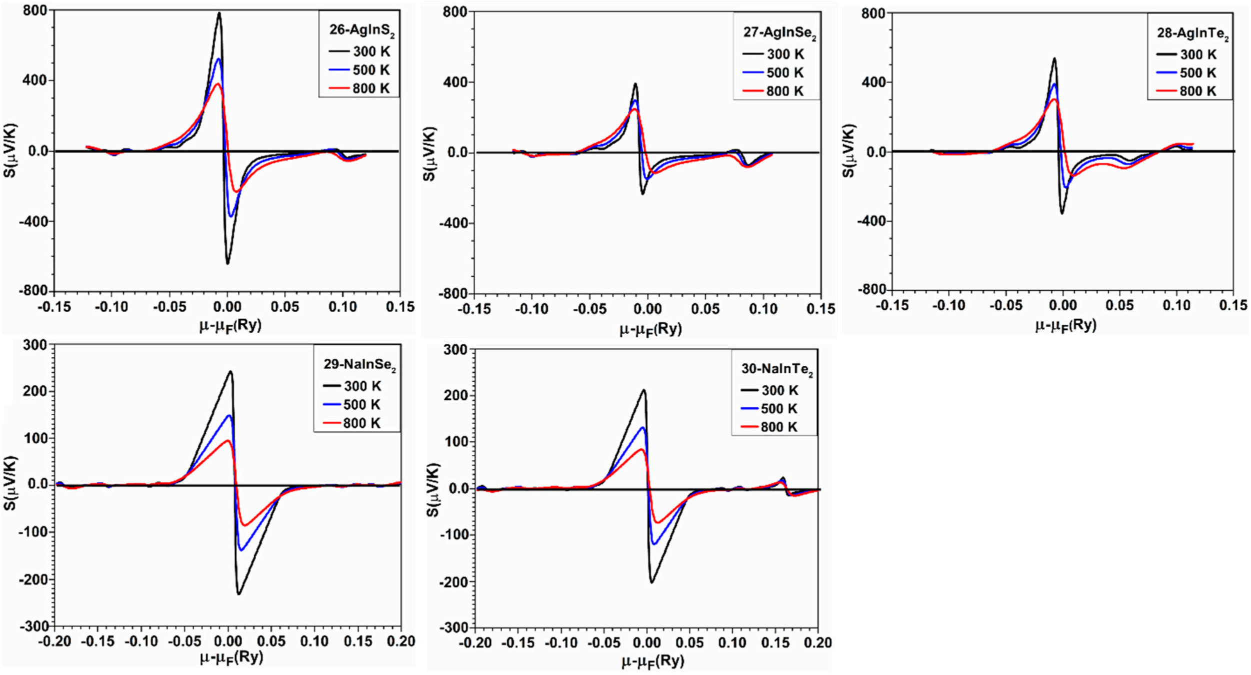

Figure S3. Calculated Seebeck coefficient(S) versus the chemical potential for the crystals from 17-$CuGaS_2$ to 30-$NaInTe_2$.

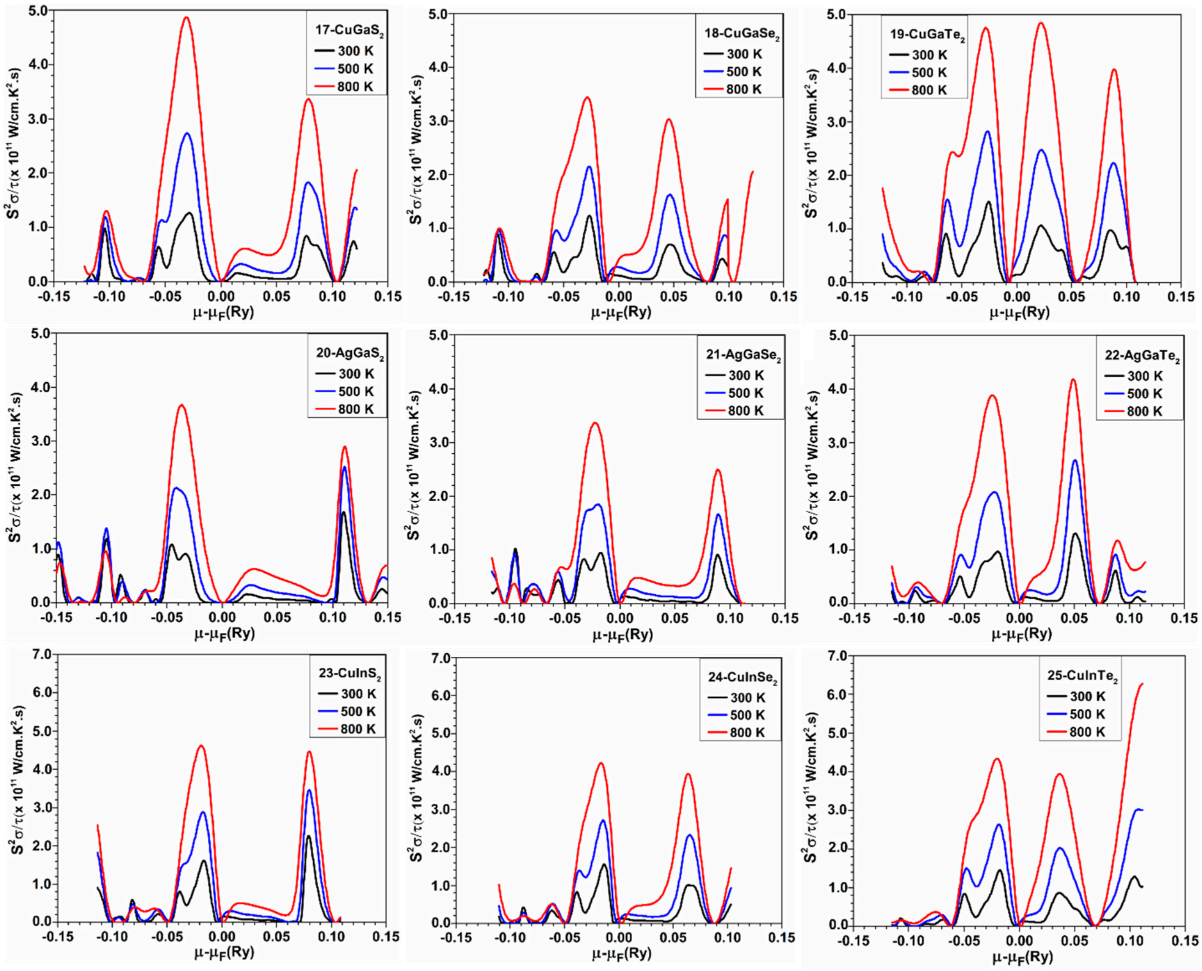

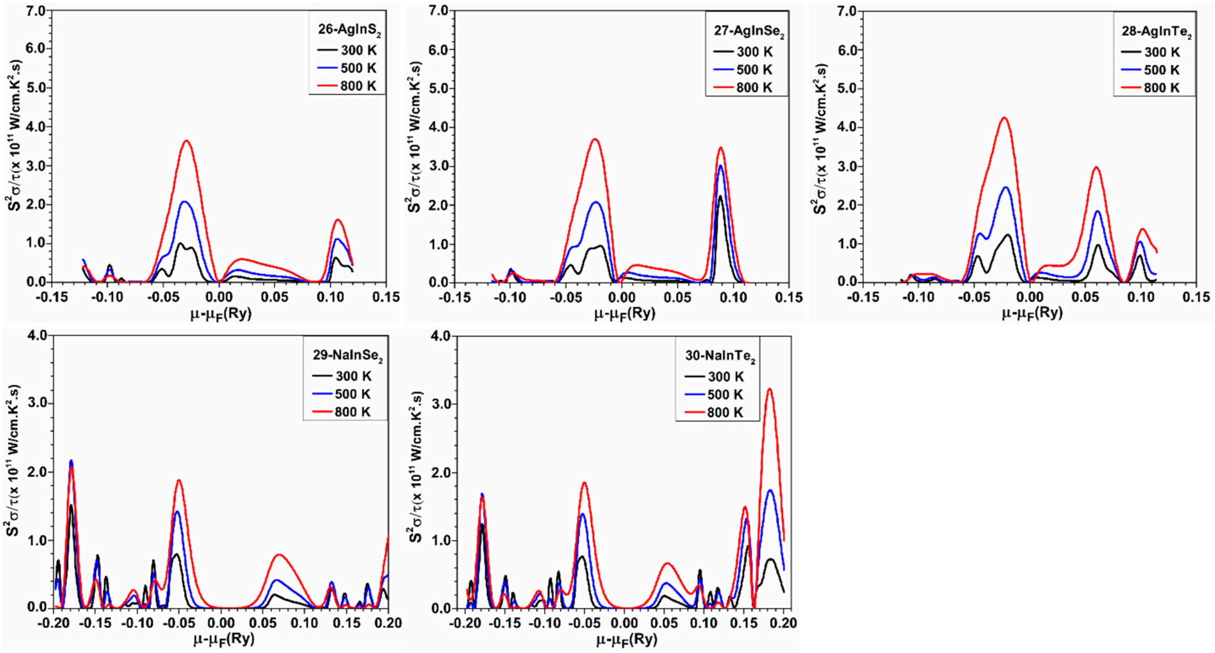

Figure S4. Calculated PF versus the chemical potential for the crystals from 17-CuGaS$_2$ to 30-NaInTe$_2$.

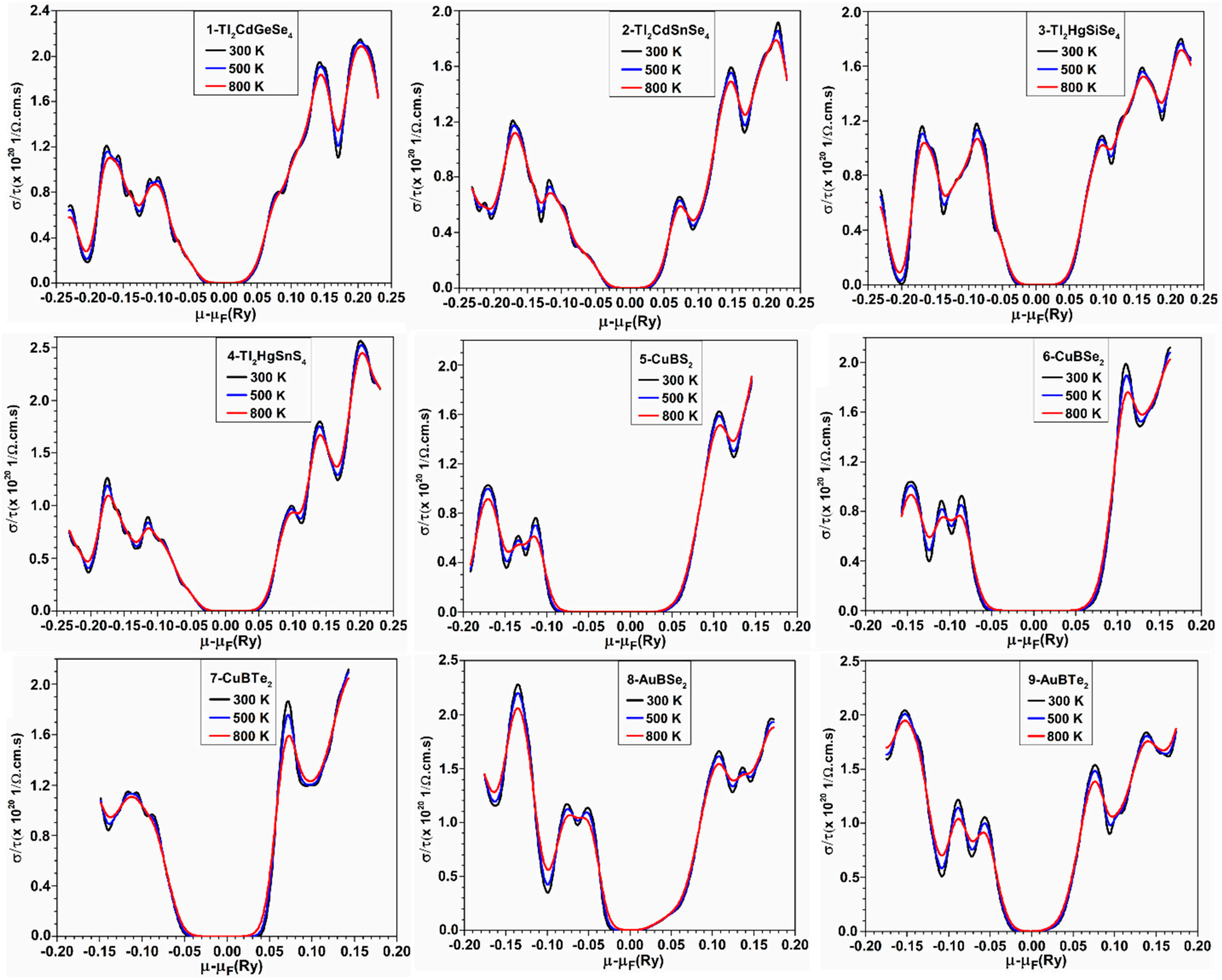

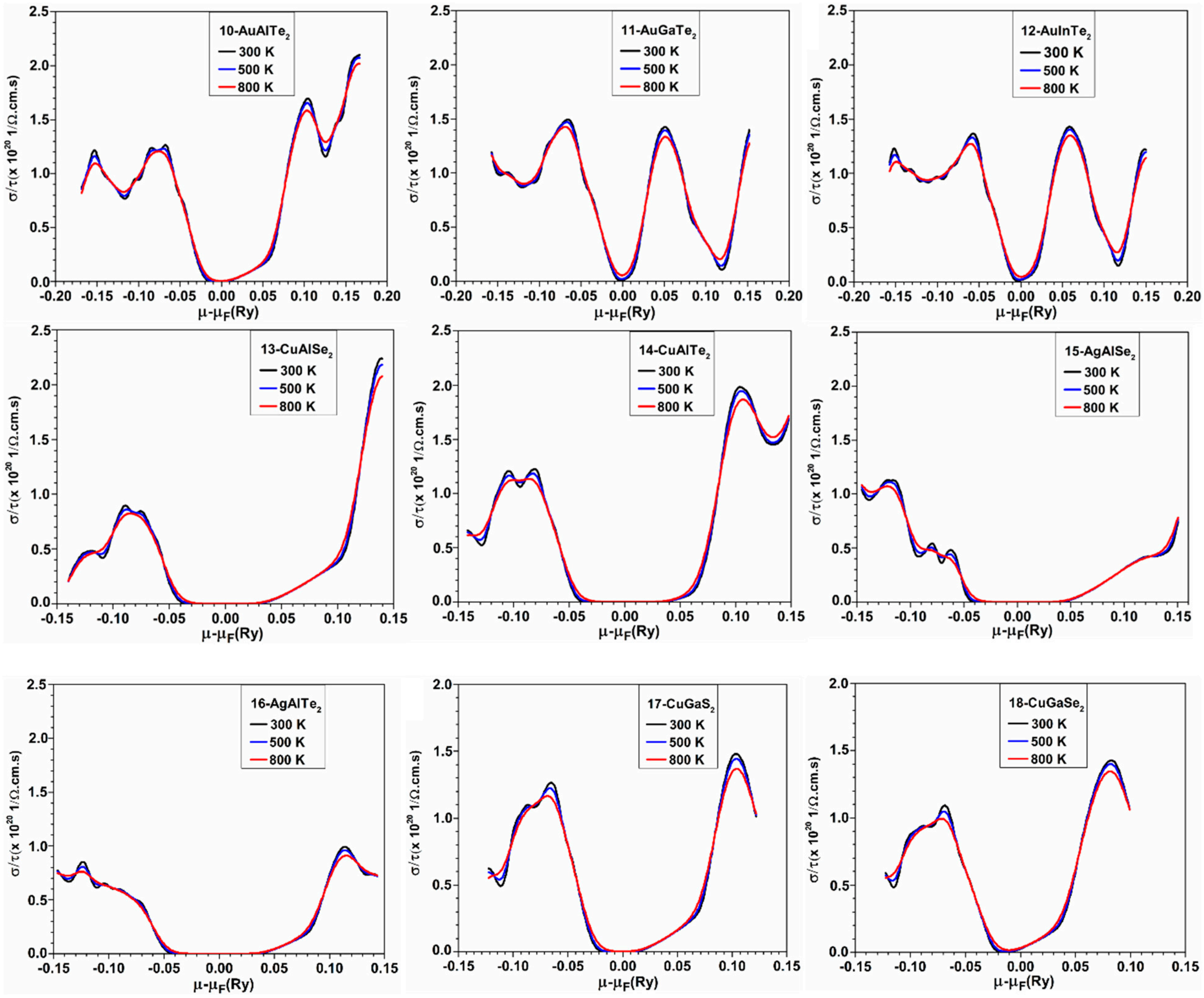
10-AuAlTe2
11-AuGaTe2
12-AuInTe2
13-CuAlSe2
14-CuAlTe2
15-AgAlSe2
16-AgAlTe2
17-CuGaS2
18-CuGaSe2
300 K
500 K
800 K
σ/τ(x 10^20 1/Ω.cm.s)
μ-μF(Ry)

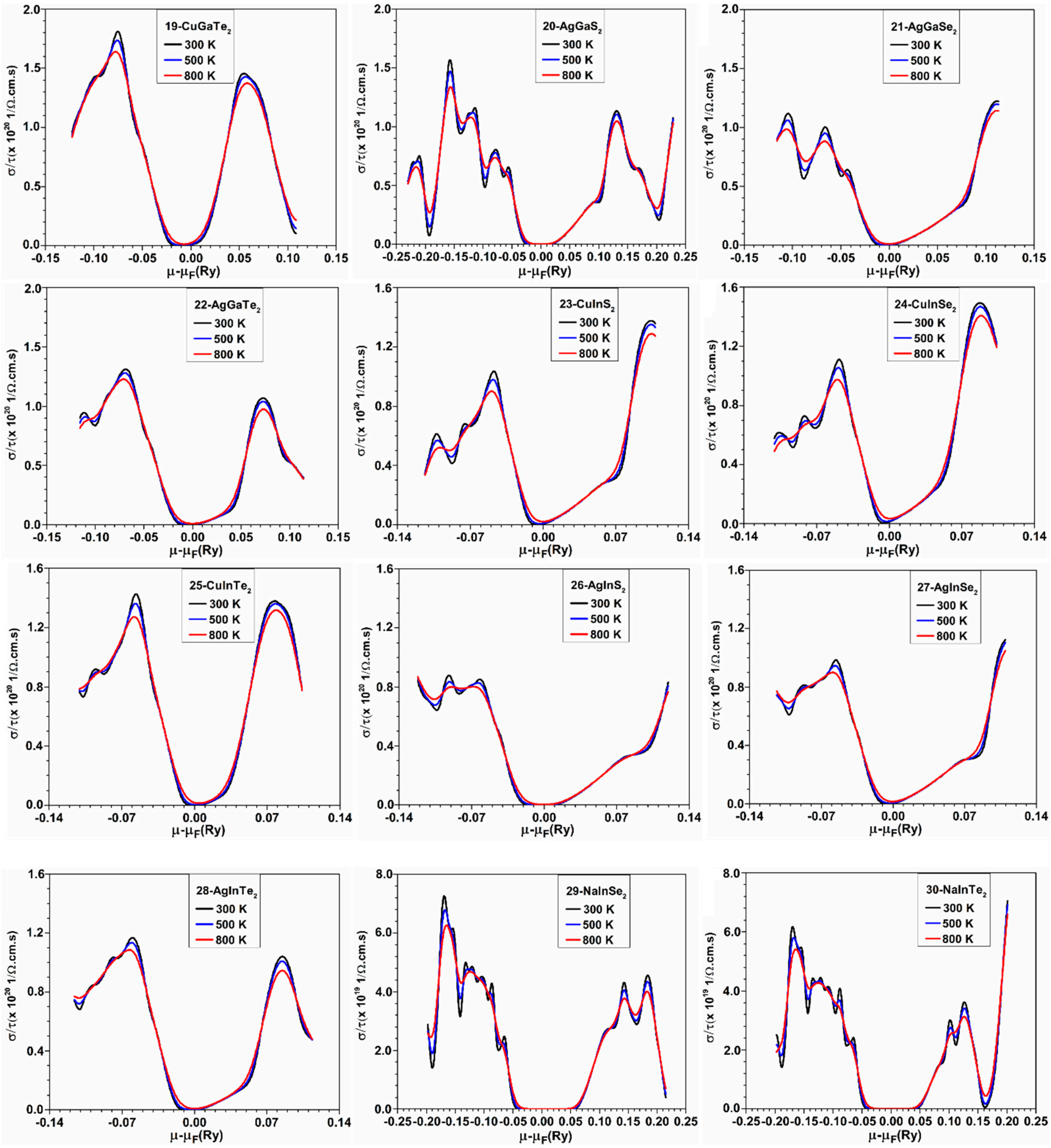

Figure S5. Calculated σ/τ versus the chemical potential for the 30 crystals.

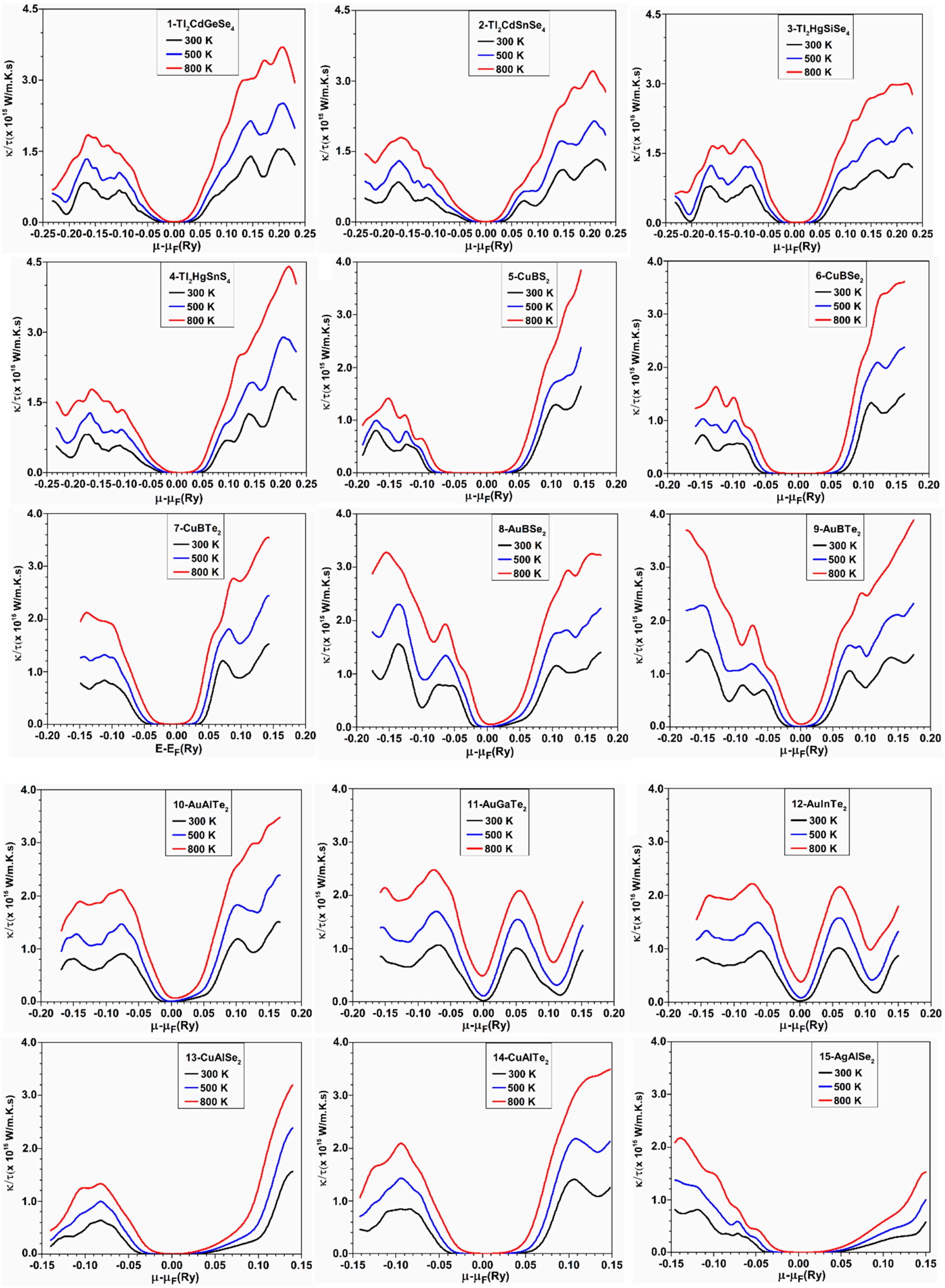
1-Tl2CdGeSe4
2-Tl2CdSnSe4
3-Tl2HgSiSe4
4-Tl2HgSnS4
5-CuBS2
6-CuBSe2
7-CuBTe2
8-AuBSe2
9-AuBTe2
10-AuAlTe2
11-AuGaTe2
12-AuInTe2
13-CuAlSe2
14-CuAlTe2
15-AgAlSe2
300 K
500 K
800 K
κ/τ(x 10^15 W/m.K.s)
κ/τ(x 10^16 W/m.K.s)
μ-μF(Ry)
E-EF(Ry)

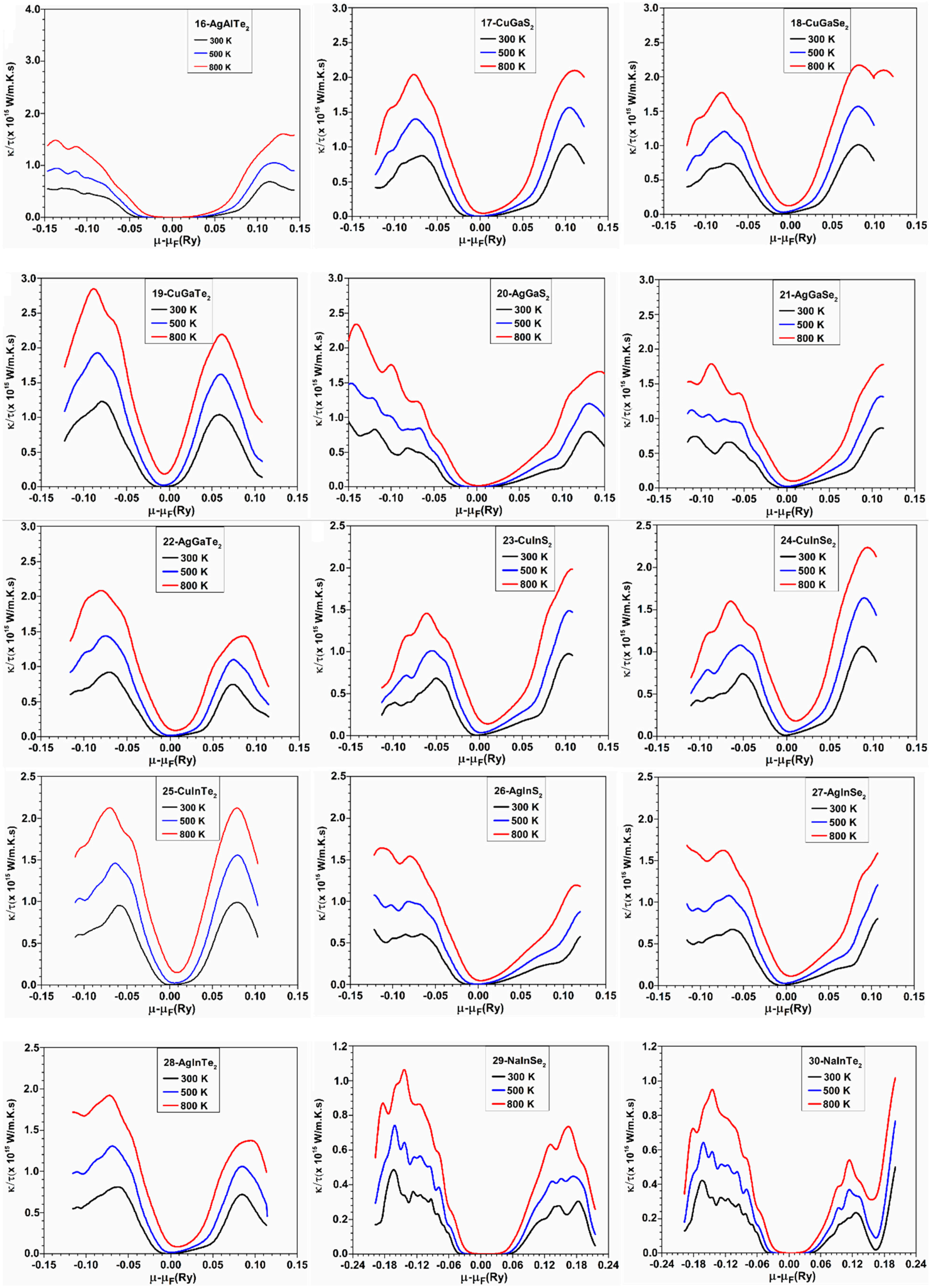

Figure S6. Calculated κ/τ versus the chemical potential for the 30 crystals.

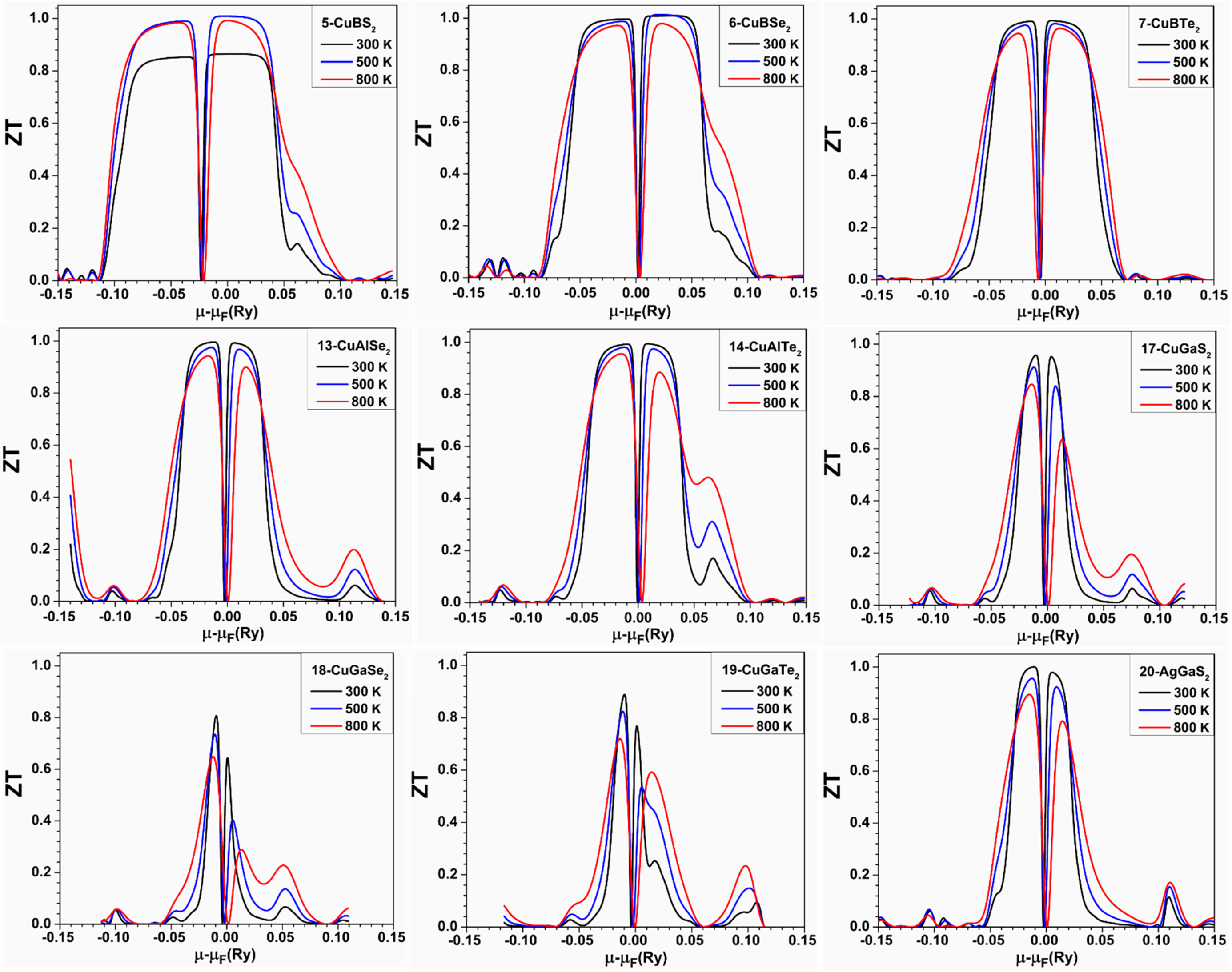
5-CuBS$_2$
6-CuBSe$_2$
7-CuBTe$_2$
13-CuAlSe$_2$
14-CuAlTe$_2$
17-CuGaS$_2$
18-CuGaSe$_2$
19-CuGaTe$_2$
20-AgGaS$_2$
300 K
500 K
800 K
ZT
μ-μ$_F$(Ry)

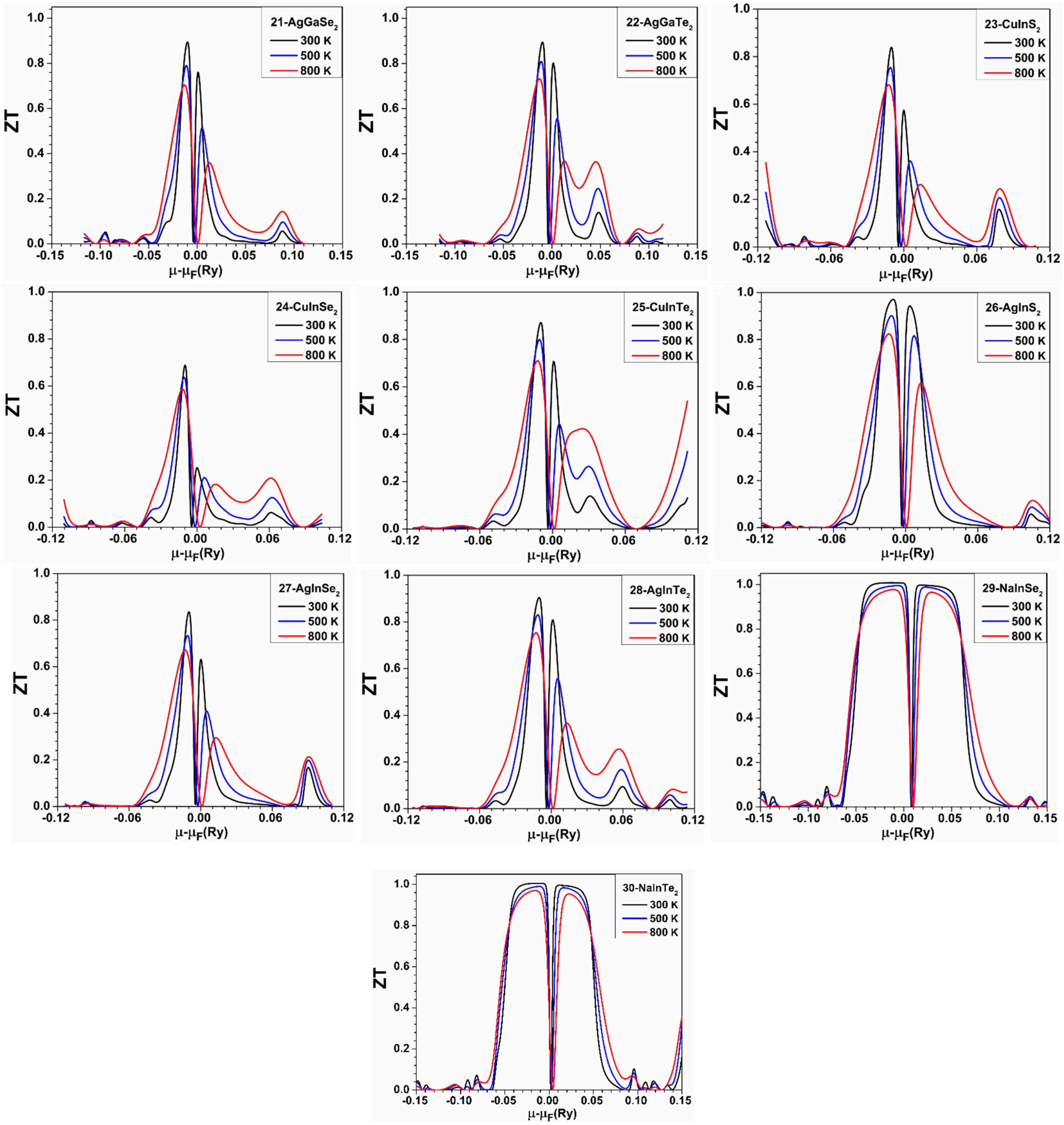

Figure S7. Calculated ZT versus the chemical potential.

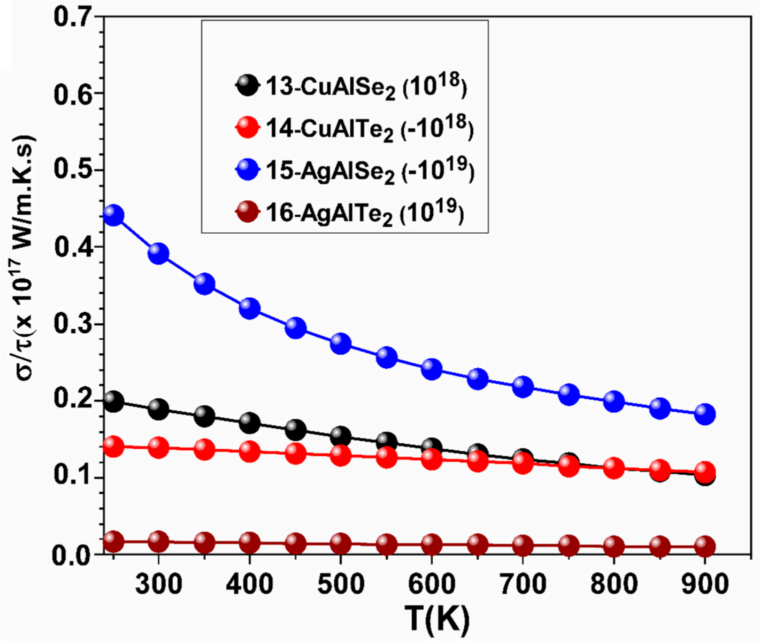

Figure S8. Calculated σ/τ versus the temperature for the crystals 13–16.

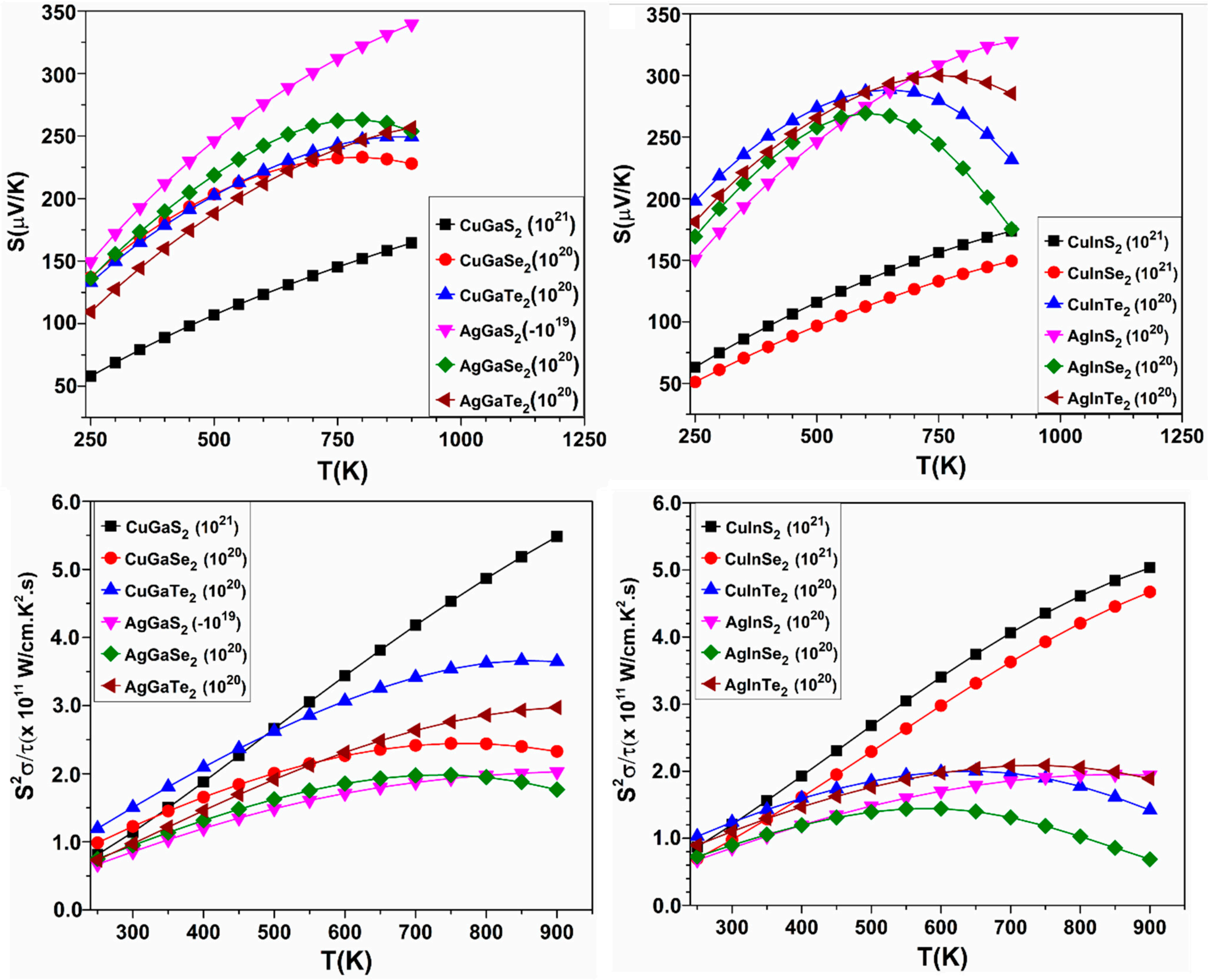

Figure S9. Calculated S and PF versus the temperature for the crystals from 17 to 30.

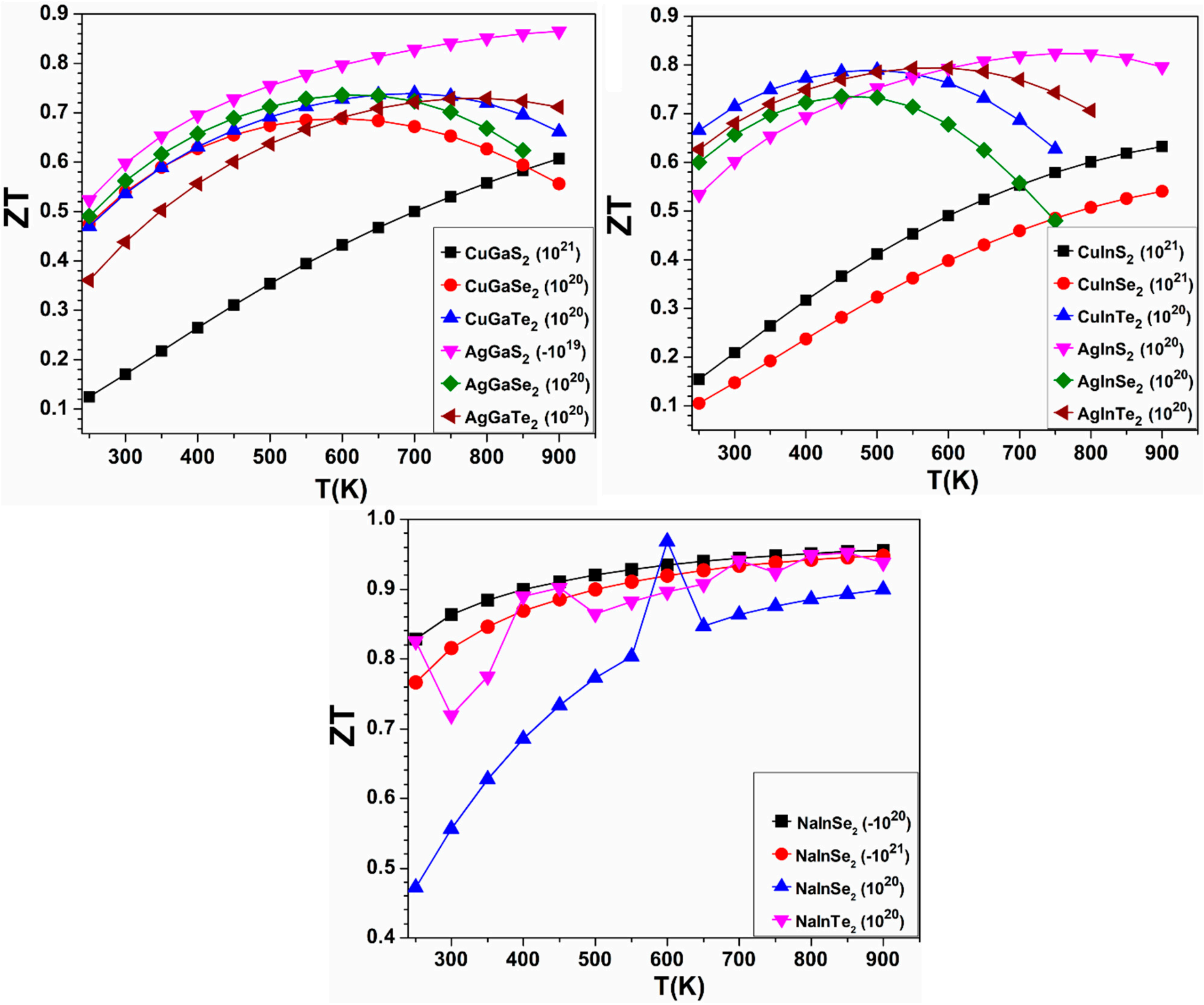

Figure S10. Calculated ZT versus the temperature for the crystals from 17 to 30.

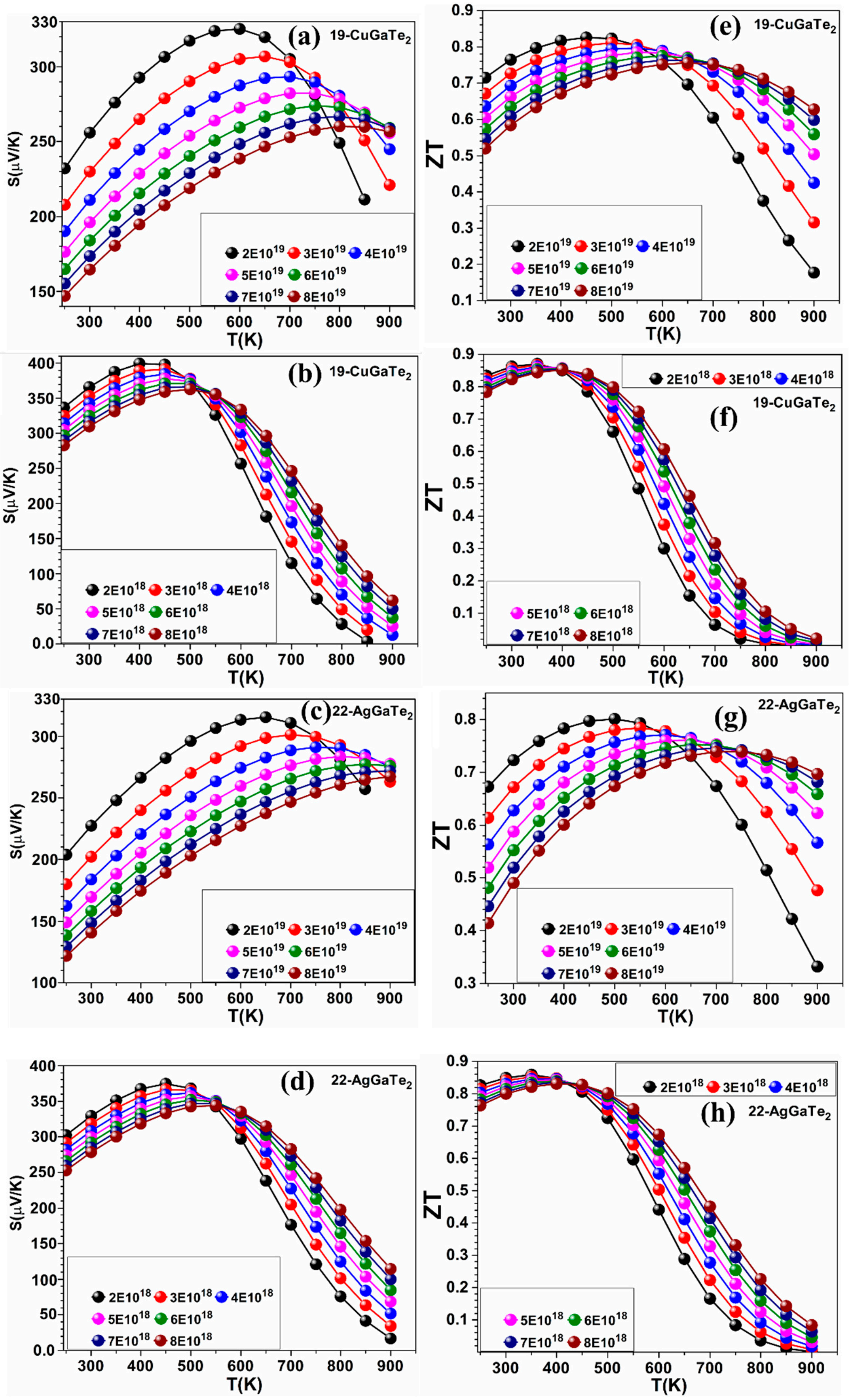

Figure S11. S and ZT versus the temperature for the crystals: (a,b,e,f) are for 19-CuGaTe2 crystal, and (c,d,g,h) are for 22-AgGaTe2 crystal at two different ranges of n.

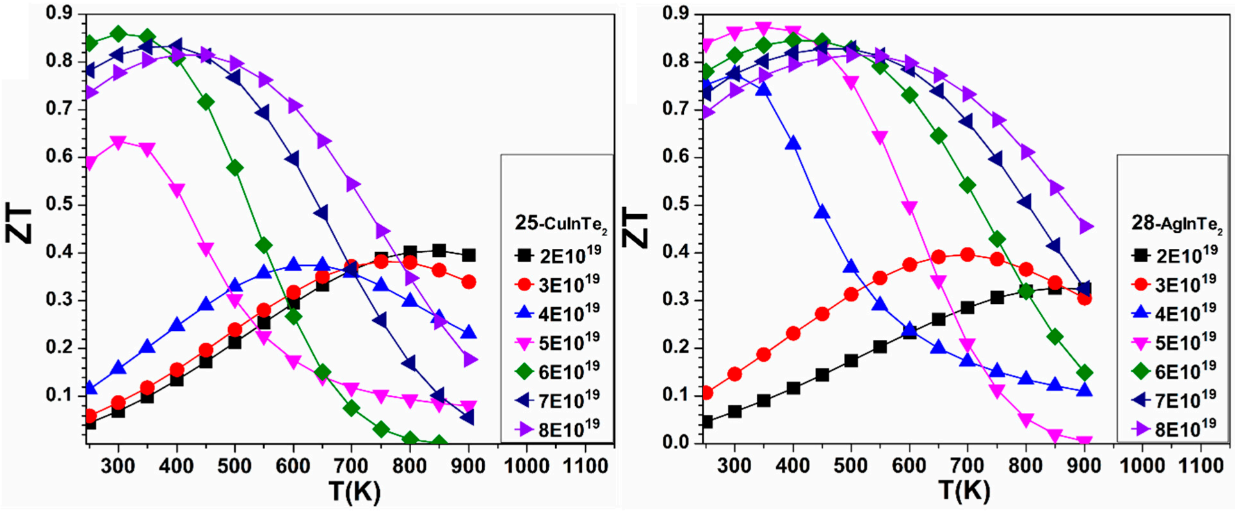

Figure S12. Shows our results for ZT versus temperature at range of n:(+2x10[19], +8x10[19] in e-/cm[3]) for the crystals 25-CuInTe2 and 28-AgInTe2

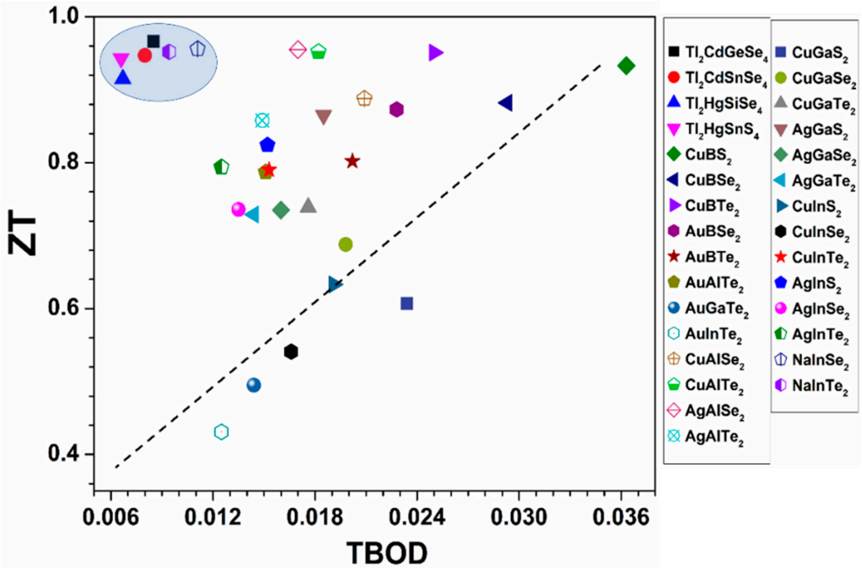

Figure S13. Calculated TBOD versus ZT for the 30 crystals.

References

1. Selezen, A.; Olekseyuk, I.; Myronchuk, G.; Smitiukh, O.; Piskach, L. Synthesis and structure of the new semiconductor compounds Tl2BIIDIVX4 (BII–Cd, Hg; DIV–Si, Ge; X–Se, Te) and isothermal sections of the Tl2Se–CdSe-Ge (Sn) Se2 systems at 570 K. *Journal of Solid State Chemistry* **2020**, 121422.
2. Mozolyuk, M.Y.; Piskach, L.; Fedorchuk, A.; Olekseyuk, I.; Parasyuk, O. Physico-chemical interaction in the Tl2Se–HgSe–DIVSe2 systems (DIV–Si, Sn). *Materials Research Bulletin* **2012**, *47*, 3830-3834.
3. Lavrentyev, A.; Gabrelian, B.; Vu, T.V.; Ananchenko, L.; Myronchuk, G.; Parasyuk, O.; Tkach, V.; Kopylova, K.; Khyzhun, O. Electronic and optical properties of quaternary sulfide Tl2HgSnS4, a promising optoelectronic semiconductor: A combined experimental and theoretical study. *Optical Materials* **2019**, *92*, 294-302.
4. Bagci, S.; Yalcin, B.G.; Aliabad, H.R.; Duman, S.; Salmankurt, B. Structural, electronic, optical, vibrational and transport properties of CuBX 2 (X= S, Se, Te) chalcopyrites. *RSC advances* **2016**, *6*, 59527-59540.
5. Yaseen, M.S.; Sun, J.; Fang, H.; Murtaza, G.; Sholl, D.S. First-principles study of electronic and optical properties of ternary compounds AuBX2 (X= S, Se, Te) and AuMTe2 (M= Al, In, Ga). *Solid State Sciences* **2021**, *111*, 106508.
6. Gudelli, V.K.; Kanchana, V.; Vaitheeswaran, G. CuAlTe2: A promising bulk thermoelectric material. *Journal of Alloys and Compounds* **2015**, *648*, 958-965.
7. Dongho Nguimdo, G.; Joubert, D.P. A density functional (PBE, PBEsol, HSE06) study of the structural, electronic and optical properties of the ternary compounds AgAlX2 (X= S, Se, Te). *The European Physical Journal B* **2015**, *88*, 1-10.

8. Asokamani, R.; Amirthakumari, R.; Rita, R.; Ravi, C. Electronic structure calculations and physical properties of ABX2 (A= Cu, Ag; B= Ga, In; X= S, Se, Te) ternary chalcopyrite systems. *physica status solidi (b)* **1999**, *213*, 349-363.
9. Yaseen, M.S.; Murtaza, G.; Murtaza, G. Theoretical investigation of the structural stabilities, optoelectronic and thermoelectric properties of ternary alloys N a I n Y 2 (Y= S, S e and T e) through modified Becke–Johnson exchange potential. *International Journal of Modern Physics B* **2020**, *34*, 2050133.
10. Yusufu, A.; Kurosaki, K.; Kosuga, A.; Sugahara, T.; Ohishi, Y.; Muta, H.; Yamanaka, S. Thermoelectric properties of Ag1− x GaTe2 with chalcopyrite structure. *Applied Physics Letters* **2011**, *99*, 061902.
11. Gui, Y.; Ye, L.; Jin, C.; Zhang, J.; Wang, Y. The nature of the high thermoelectric properties of CuInX2 (X= S, Se and Te): First-principles study. *Applied Surface Science* **2018**, *458*, 564-571.
12. Kucek, V.; Drasar, C.; Navratil, J.; Plechacek, T.; Benes, L. Thermoelectric properties of Ni-doped CuInTe2. *Journal of Physics and Chemistry of Solids* **2015**, *83*, 18-23.
13. Yang, J.; Fan, Q.; Cheng, X. Prediction for electronic, vibrational and thermoelectric properties of chalcopyrite AgX (X= In, Ga) Te2: PBE+ U approach. *Royal Society open science* **2017**, *4*, 170750.
14. Aikebaier, Y.; Kurosaki, K.; Sugahara, T.; Ohishi, Y.; Muta, H.; Yamanaka, S. High-temperature thermoelectric properties of non-stoichiometric Ag1−xInTe2 with chalcopyrite structure. *Materials Science and Engineering: B* **2012**, *177*, 999-1002.
